# Central Role of Hypothalamic Circuits for Acupuncture's Anti‐Parkinsonian Effects

**DOI:** 10.1002/advs.202403245

**Published:** 2024-08-09

**Authors:** Ju‐Young Oh, Hyowon Lee, Sun‐Young Jang, Hyunjin Kim, Geunhong Park, Almas Serikov, Jae‐Hwan Jang, Junyeop Kim, Seulkee Yang, Moonsun Sa, Sung Eun Lee, Young‐Eun Han, Tae‐Yeon Hwang, Sharon Jiyoon Jung, Hee Young Kim, Seung Eun Lee, Soo‐Jin Oh, Jeongjin Kim, Jeongyeon Kim, Jongpil Kim, Thomas J. McHugh, C. Justin Lee, Min‐Ho Nam, Hi‐Joon Park

**Affiliations:** ^1^ College of Korean Medicine Kyung Hee University Seoul 02447 Republic of Korea; ^2^ Studies of Translational Acupuncture Research (STAR) Kyung Hee University Seoul 02447 Republic of Korea; ^3^ Brain Science Institute Korea Institute of Science and Technology (KIST) Seoul 02792 Republic of Korea; ^4^ Department of KHU‐KIST Convergence Science and Technology Kyung Hee University Seoul 02447 Republic of Korea; ^5^ Laboratory of Stem Cells & Cell Reprogramming Department of Chemistry Dongguk University Seoul 04629 Republic of Korea; ^6^ Center for Cognition and Sociality Institute for Basic Science Daejeon 34126 Republic of Korea; ^7^ Technological Convergence Center Korea Institute of Science and Technology (KIST) Seoul 02792 Republic of Korea; ^8^ Department of Physiology Yonsei University Seoul 03722 Republic of Korea; ^9^ Research Animal Resource Center Korea Institute of Science and Technology (KIST) Seoul 02792 Republic of Korea; ^10^ Emotion, Cognition & Behavior Research Group Korea Brain Research Institute Daegu 41062 Republic of Korea; ^11^ Laboratory for Circuit and Behavioral Physiology RIKEN Wako‐shi Saitama 351‐0198 Japan

**Keywords:** acupuncture, hypothalamus, melanin‐concentrating hormone (MCH), motor and non‐motor symptoms, neural circuitry, Parkinson's disease (PD)

## Abstract

Despite clinical data stretching over millennia, the neurobiological basis of the effectiveness of acupuncture in treating diseases of the central nervous system has remained elusive. Here, using an established model of acupuncture treatment in Parkinson's disease (PD) model mice, we show that peripheral acupuncture stimulation activates hypothalamic melanin‐concentrating hormone (MCH) neurons via nerve conduction. We further identify two separate neural pathways originating from anatomically and electrophysiologically distinct MCH neuronal subpopulations, projecting to the substantia nigra and hippocampus, respectively. Through chemogenetic manipulation specifically targeting these MCH projections, their respective roles in mediating the acupuncture‐induced motor recovery and memory improvements following PD onset are demonstrated, as well as the underlying mechanisms mediating recovery from dopaminergic neurodegeneration, reactive gliosis, and impaired hippocampal synaptic plasticity. Collectively, these MCH neurons constitute not only a circuit‐based explanation for the therapeutic effectiveness of traditional acupuncture, but also a potential cellular target for treating both motor and non‐motor PD symptoms.

## Graphical abstract

Peripheral acupuncture stimulation activates hypothalamic MCH neurons, leading to motor recovery and memory improvement in Parkinson's disease model mice. This study reveals distinct neural pathways from MCH neurons to the substantia nigra and hippocampus, elucidating the mechanisms of acupuncture‐induced recovery from dopaminergic neurodegeneration and hippocampal synaptic plasticity impairment, offering insights into therapeutic targets for PD.

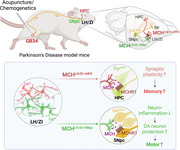

## 1 Introduction

Parkinson's disease (PD) is a second‐most common neurodegenerative disorder whose prevalence is known to rise with age, impacting 1% of people over the age of 60.^[^ ^1^ ^]^ PD is characterized by typical movement symptoms, such as resting tremors, bradykinesia, rigidity, and postural instability which is caused by extensive death or dysfunction of dopaminergic neurons in the substantia nigra pars compacta (SNpc). Additionally, PD is well known to be accompanied by noticeable non‐motor symptoms, including cognitive impairment and mental disorders.^[^ ^2^ ^]^ Although current PD treatments, such as levodopa and deep brain stimulation (DBS), have demonstrated therapeutic effects on parkinsonian motor symptoms in clinics,^[^ ^2^ ^]^ they have limitations. Long‐term use of levodopa frequently leads to levodopa‐induced dyskinesia (LID), and DBS is difficult to apply in patients with early‐stage PD with mild symptoms due to its high invasiveness. Furthermore, most current therapies primarily address motor dysfunctions,^[^ ^2^ ^]^ leaving out non‐motor symptoms, such as memory deficits, which significantly impact the patients’ quality of life. As a result, PD remains as a complex, devastating, and irreversible neurodegenerative disorder. Therefore, an alternative, safe, and less invasive therapeutic approach that addresses both motor and memory deficits is required.

In East Asia, acupuncture, a traditional form of medicine, has been clinically utilized for treating various neurological conditions such as PD for several thousands of years.^[^ ^3^ ^]^ Acupuncture involves the insertion and manipulation, including lifting‐thrusting and twisting‐rotating, of thin needles into specific peripheral acupoints in the skin and sometimes the muscular layer.^[^ ^3^, ^4^ ^]^ It has been clinically applied to regulate homeostasis, leading to improvements in both motor and non‐motor symptoms in PD. Specifically, an acupoint located on the hindlimb, GB34 *(Yanglingquan*), has been noted for its potential to alleviate PD symptoms in patients,^[^ ^5^ ^]^ suggesting that acupuncture could serve as an alternative therapy for PD. Particularly, functional magnetic resonance imaging studies have shown that acupuncture at GB34 activates specific brain regions involved in motor control, such as the precentral gyrus and putamen in both healthy subjects^[^ ^6^ ^]^ and PD patients.^[^ ^7^ ^]^ Preclinical studies have also indicated that acupuncture at GB34 can enhance synaptic dopamine availability,^[^ ^8^ ^]^ increase neuroprotective agents such as brain‐derived neurotrophic factors (BDNF) and cyclophilin A,^[^ ^9^ ^]^ and reduce inflammation and oxidative stress^[^ ^9^, ^10^ ^]^ in the SNpc and the striatum of PD animal models.^[^ ^11^ ^]^ However, a circuit‐based mechanistic understanding underlying these anti‐parkinsonian effects of acupuncture remains unknown. Only recently have neuroscientists begun to make significant progress in understanding the neurobiological basis of acupuncture's effects, particularly its systemic anti‐inflammatory effects, which have been primarily investigated at the peripheral and spinal levels.^[^ ^4^, ^12^ ^]^ Therefore, unraveling the neurobiological mechanism of acupuncture's anti‐parkinsonian effects demands immediate attention.

We previously raised the possibility that acupuncture stimulation at GB34 in PD mouse models could potentially activate diencephalic melanin‐concentrating hormone (MCH) neurons.^[^ ^13^ ^]^ In the same study, we also suggested that MCH could have a neuroprotective role against PD.^[^ ^13^ ^]^ MCH neurons are specifically localized in the lateral hypothalamus (LH) and zona incerta (ZI) regions.^[^ ^14^ ^]^ The LH is implicated in PD pathophysiology, as evidenced by accumulation of Lewy bodies and loss of several types of neurons including MCH neurons.^[^ ^15^ ^]^ The ZI has been known for its potential role in controlling basal ganglia circuits.^[^ ^16^ ^]^ However, a critical question has remained unanswered: whether and how the MCH^LH/ZI^ neurons mediate the effect of acupuncture on PD‐related symptoms. The projections of MCH neurons to the hippocampus (HPC) and their physiological roles in sleep, mood, and memory have been well documented,^[^ ^17^ ^]^ which inspired us to investigate the possible role of MCH neurons in non‐motor deficits, such as memory impairment in PD. On the other hand, the involvement of MCH neurons and the related neural circuits in motor control has been scarcely investigated. Only one study has provided anatomical evidence for existence of MCH neuronal projection to the SNpc, but without functional investigation.^[^ ^18^ ^]^

The aim of this study was to investigate the molecular, cellular, and circuit‐level mechanisms underlying the effect of MCH neuron‐mediated acupuncture on motor and non‐motor phenotypes in PD. By adopting a multidisciplinary approach, including in‐vivo real‐time and *ex‐vivo* Ca^2+^ imaging, retrograde viral tracing, light‐sheet fluorescent microscopy, electrophysiology, cell type‐specific and projection‐specific chemogenetic manipulation, and RNA‐sequencing, we tested the hypothesis that acupuncture stimulation enhances MCH neuronal activity and, in turn, elicits alleviating effects on PD‐like motor and memory deficits via the novel MCH^LH/ZI→SNpc^ projection and the well‐established MCH^LH→HPC^ projections,^[^ ^17^, ^19^ ^]^ respectively. By elucidating the underlying mechanisms of acupuncture's effects on PD, this study could facilitate the development of targeted, evidence‐based acupuncture protocols that improve both motor and non‐motor symptoms in PD patients.

## 2 Results

### 2.1 Acupuncture Alleviates PD‐like Motor and Memory Deficits through Nerve Conduction

**Figure 1:**
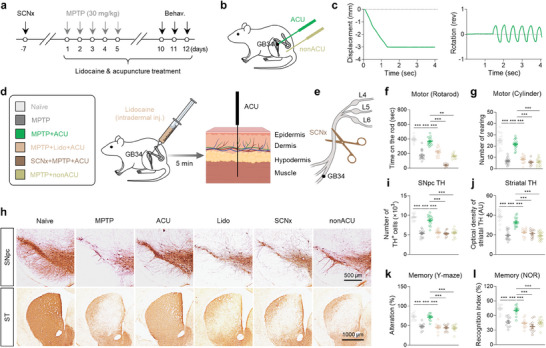
Acupuncture stimulation at GB34 elicits anti‐parkinsonian effects through nerve conduction. a) Experimental timeline of various interventions in the MPTP mouse model. b) Schematic diagram of acupuncture treatment at GB34 acupoint and non‐acupoint. c) Quantitative measurement of displacement and rotation of the needle during acupuncture stimulation. d) Left, group information. Right, schematic diagram of lidocaine‐induced local nerve blockade 5 min prior to acupuncture stimulation. e) Schematic diagram of sciatic nerve axotomy 7 days prior to acupuncture stimulation. f,g) Motor function assessed by rotarod test (f) and cylinder test g,h) Representative images of TH staining with SNpc and striatum (ST) tissues. i) Numbers of TH‐positive dopaminergic neurons in the SNpc. j) Quantification of optical density of striatal TH. k,l) Spatial working memory function assessed by Y‐maze k) and novel object recognition (NOR) test l). Statistical significance was assessed by Kruskal‐Wallis ANOVA test with Dunn's multiple comparison test f) or one‐way ANOVA with Tukey's multiple comparison test. ^**^ *p* < 0.01. ^***^ *p* < 0.001. All data are presented as mean ± SEM. Detailed statistical information is listed in Table S1 (Supporting Information).

We first assessed whether acupuncture stimulation alleviates the motor and memory deficits exhibited in the 1‐Methyl‐4‐phenyl‐1,2,3,6‐tetrahydropyridine (MPTP)‐induced subchronic PD mouse model,^[^ ^20^ ^]^ (**Figure** **1**a). Acupuncture stimulation was performed by a bilateral needle insertion into the hindlimb GB34 acupoint or a control non‐acupoint to a depth of 3 mm and rotation for 30 s at a rate of two spins per second without anesthesia (Figure 1b,c), as previously described.^[^ ^8^, ^9^, ^13^, ^21^ ^]^ We assessed the effects of acupuncture stimulation at GB34 by comparing it with an untreated MPTP mouse model (MPTP) and a non‐acupoint stimulated MPTP mouse model (MPTP + nonACU) (Figure 1d). Consistent with previous findings,^[^ ^13^, ^21^ ^]^ we observed that acupuncture stimulation at GB34 alleviated the motor dysfunction assessed by the rotarod and cylinder tests (Figure 1f,g), and mitigated the TH‐positive cell loss in the SNpc (Figure 1h,i; Figure S1a, Supporting Information), reduction in the optical density of striatal TH (Figure 1h,j) in MPTP‐induced PD mouse model. In contrast, these effects were not observed with non‐acupoint stimulation (Figure 1f–j). Additionally, by performing Y‐maze and novel object recognition (NOR) tests, we found that acupuncture treatment significantly alleviated MPTP‐induced memory deficits, while non‐acupoint stimulation did not (Figure 1k,l). The 7‐day acupuncture treatment that started after the completion of MPTP administration also exhibited partial, but significant anti‐parkinsonian effects (Figure S1b–i, Supporting Information). Taken together, acupuncture stimulation significantly alleviated both the PD‐like motor and memory deficits in the MPTP mouse model.

Based on recent findings about the possible involvement of afferent nerve conduction in acupuncture effects,^[^ ^4^, ^12^ ^]^ we postulated that periphery‐to‐brain nerve conduction could mediate the anti‐parkinsonian effects of acupuncture stimulation at GB34. To examine the necessity of this mechanism, we performed lidocaine‐induced local nerve block (7 mg kg^−1^; Figure 1d) or axotomy of the sciatic nerve before acupuncture at GB34 (Figure 1e). We found that both lidocaine application and sciatic nerve axotomy significantly blocked all observed anti‐parkinsonian effects of acupuncture in the MPTP model (Figure 1f–l), suggesting that sensory nerve conduction from the peripheral acupoint is necessary for the effects of acupuncture stimulation at GB34. These findings indicate that activation of the afferent nerve endings at GB34 is necessary to mimic the effects of acupuncture.

### 2.2 Anatomical and Functional Connection between Peripheral Acupoint and Central MCH^LH/ZI^ Neurons

**Figure 2:**
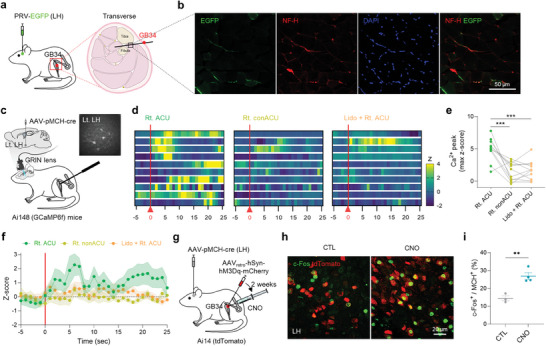
Anatomical and functional connection between a peripheral acupoint GB34 and MCH^LH/ZI^ neurons. a) Schematic diagram of retrograde tracing of the neural path from the LH to the peripheral acupoint GB34 using pseudorabies virus (PRV). b) Representative fluorescence image of EGFP‐labeled nerve endings (stained with neurofilament NF‐H) at the muscular layer of GB34. c) Schematic diagram of in‐vivo calcium imaging of MCH^LH/ZI^ neurons upon acupuncture stimulation. Right top, an example image of MCH‐neuronal *GCaMP6f* signals. d) Heatmaps of calcium signals from each neuron upon acupuncture or control stimulations. e) Quantification of peak amplitudes of calcium signals. f) Averaged time course of relative changes in *GCaMP6f* fluorescence indicating calcium signals. The average per group of the Z scored data is shown. g) Schematic diagram of chemogenetic stimulation of peripheral afferent nerve fibers at acupoint GB34. h) Representative confocal images of c‐Fos expression in the MCH^LH/ZI^ neurons 1 h after chemogenetic stimulation of acupoint GB34. i) Quantification of c‐Fos^+^ MCH^LH/ZI^ neurons upon chemogenetic stimulation of acupoint GB34. Statistical significance was assessed by repeated‐measure one‐way ANOVA with Tukey's multiple comparison test e) or two‐tailed unpaired t‐test. ^**^ *p* < 0.01. ^***^ *p* < 0.001. All data are presented as mean ± SEM. Detailed statistical information is listed in Table S1 (Supporting Information).

Based on previous studies demonstrating that the hypothalamic area, particularly LH, is an important mediator of the effects of acupuncture,^[^ ^13^, ^22^ ^]^ we aimed to investigate whether there exists an anatomical connection between acupoint GB34 and the LH. We utilized a retrograde tracing approach, employing pseudorabies virus (PRV) expressing enhanced green fluorescent protein (EGFP) to label neural pathways entering into the LH. This tracing revealed rare but distinct GFP‐labeled nerve endings in the muscular layers at the peripheral acupoint GB34 (**Figure** **2**a,b). Furthermore, by conducting bidirectional retrograde tracing from both GB34 and the LH/ZI region, we confirmed the presence of neural connections from GB34 to the LH/ZI region. (Figure 2a; Figure S2a, Supporting Information).

Subsequently, our focus shifted toward examining the functional link between acupoint GB34 and MCH^LH/ZI^ neurons, given the previous evidence suggesting an increase in MCH levels following acupuncture stimulation at GB34.^[^ ^13a^ ^]^ To explicitly assess the functional connectivity between GB34 and MCH^LH/ZI^ neurons, we performed in‐vivo Ca^2+^ imaging using single‐photon microendoscopy upon acupuncture at GB34 in head‐fixed and lightly anesthetized mice. We utilized Ai148 mice, which Cre‐dependently express GCaMP6f,^[^ ^23^ ^]^ a genetically encoded calcium indicator,^[^ ^24^ ^]^ with viral expression of Cre under the pro‐MCH (pMCH)‐specific (>90%, Figure S2b, Supporting Information) promoter (AAV_DJ_‐pMCH‐Cre) in the LH/ZI region (Figure 2d). We found that acupuncture stimulation at GB34 elicited a significant Ca^2+^ rise in contralateral MCH^LH/ZI^ neurons, whereas needling at a non‐acupoint or acupoint on the opposite hindlimb did not (Figure 2c–f; Figure S2c–e, Supporting Information), implying a functional connectivity between peripheral acupoint GB34 and MCH^LH/ZI^ neurons in the brain. This result was supported by our finding that chemogenetic activation of afferent fibers at the GB34 led to a significant increase in c‐Fos expression within the MCH^LH/ZI^ neurons (Figure 2g–i). Together, these findings provided strong evidence of the anatomical and functional link between acupoint GB34 and MCH^LH/ZI^ neurons.

### 2.3 MCH^LH/ZI^ Neurons Mediate Anti‐Parkinsonian Effects of Acupuncture

**Figure 3:**
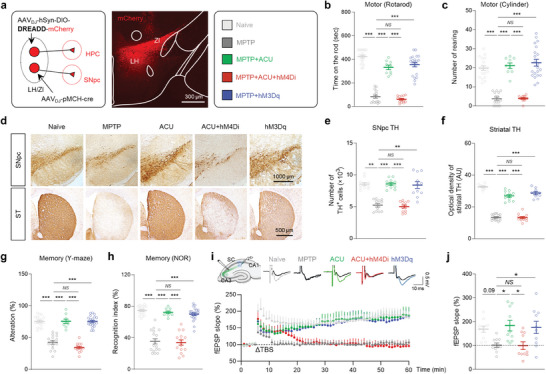
MCH^LH/ZI^ neuronal activity is critical for anti‐parkinsonian effects of acupuncture. a) Left, Schematic diagram of viral strategy for chemogenetic modulation of MCH^LH/ZI^ neurons. Middle, representative confocal image of *mCherry* expression in LH/ZI region. Right, group information. b,c) Motor function assessed by rotarod tests b) and cylinder test c) upon chemogenetic manipulation of MCH^LH/ZI^ neurons with or without acupuncture treatment in the MPTP model. d) Representative images of TH staining with SNpc and striatum (ST) tissues of each group. e) Numbers of TH‐positive dopaminergic neurons in the SNpc. f) Quantification of optical density of striatal TH. g,h) Memory function assessed by Y‐maze test g) and NOR test h) upon chemogenetic manipulation of MCH^LH/ZI^ neurons with or without acupuncture treatment in the MPTP model. i) Top, Representative fEPSP traces before (black) and 55 min after theta‐burst stimulation (TBS). Bottom, time‐course of fEPSP slope change. j) Quantification of changes in the fEPSP slopes after TBS (for the last 10 min). Statistical significance was assessed by Kruskal‐Wallis ANOVA test with Dunn's multiple comparison test e) or one‐way ANOVA with Tukey's multiple comparison test. ^**^ *p* < 0.01. ^***^ *p* < 0.001. All data are presented as mean ± SEM. Detailed statistical information is listed in Table S1 (Supporting Information).

To determine whether MCH^LH/ZI^ neurons are necessary for the anti‐parkinsonian effects of acupuncture, we adopted a chemogenetic approach to selectively inhibit MCH^LH/ZI^ neurons using hM4Di, a Gi‐coupled designer receptor exclusively activated by designer drug (DREADD)^[^ ^25^ ^]^ driven by the pMCH promoter, with systemic administration of clozapine N‐oxide (CNO, 1 mg kg^−1^) (**Figure** **3**a; Figure S3a, Supporting Information). We found that while acupuncture treatment significantly recovered motor dysfunction, as assessed by the rotarod and cylinder tests, the hM4Di‐mediated chemogenetic inhibition of MCH^LH/ZI^ neuronal activity significantly blocked the acupuncture‐mediated motor functional recovery (Figure 3b,c). Consistently, the chemogenetic inhibition of MCH^LH/ZI^ neurons significantly and fully blocked the acupuncture effects in rescuing the number of SNpc TH‐positive neurons and the striatal TH level (Figure 3d–f). Additionally, while acupuncture stimulation significantly hindered the MPTP‐induced memory deficits, as assessed by the Y‐maze and NOR tests, the chemogenetic inhibition of MCH^LH/ZI^ neurons significantly blocked the acupuncture effects (Figure 3g,h). The MPTP‐induced spatial memory deficits were associated with impaired long‐term potentiation (LTP) at the Shaffer Collateral‐CA1 synapse of the HPC (Figure 3i,j). And acupuncture treatment significantly reversed the LTP impairment, which was blocked by the chemogenetic inhibition of MCH^LH/ZI^ neurons. These findings indicate that MCH^LH/ZI^ neuronal activity is necessary for the therapeutic effects of acupuncture on both motor and non‐motor symptoms.

Next, we investigated whether MCH neurons are sufficient for the acupuncture effects by adopting hM3Dq‐mediated chemogenetic activation (Figure 3a; Figure S3b, Supporting Information). We found that the chemogenetic activation of MCH^LH/ZI^ neurons, without acupuncture, significantly prevented MPTP‐induced motor symptoms (Figure 3b,c), nigrostriatal TH loss (Figure 3e,f), and memory deficits (Figure 3g–j). These anti‐parkinsonian effects of chemogenetic activation of MCH^LH/ZI^ neurons successfully mimicked the acupuncture's effects, indicating that boosting MCH^LH/ZI^ neuronal activity is sufficient for the anti‐parkinsonian effects observed with acupuncture in the MPTP mouse model. Additionally, we tested the role of MCH^LH/ZI^ neuronal activity in the A53T‐mutated alpha‐synuclein model^[^ ^26^ ^]^ and found that chemogenetic activation of MCH^LH/ZI^ neurons significantly recovered the alpha‐synuclein‐associated motor dysfunction, memory deficits, and nigrostriatal TH loss (Figure S3c–k, Supporting Information). MCH neuronal activation also alleviated MPTP‐induced weight loss (Figure S3l–n, Supporting Information), consistent with previous findings.^[^ ^27^ ^]^ Taken together, these findings indicate that MCH^LH/ZI^ neurons are necessary and sufficient for acupuncture‐mediated alleviation of both PD‐related motor and memory deficits.

### 2.4 Two Discrete Subpopulations of MCH Neurons: MCH^LH/ZI→SNpc^ and MCH^LH→HPC^

**Figure 4:**
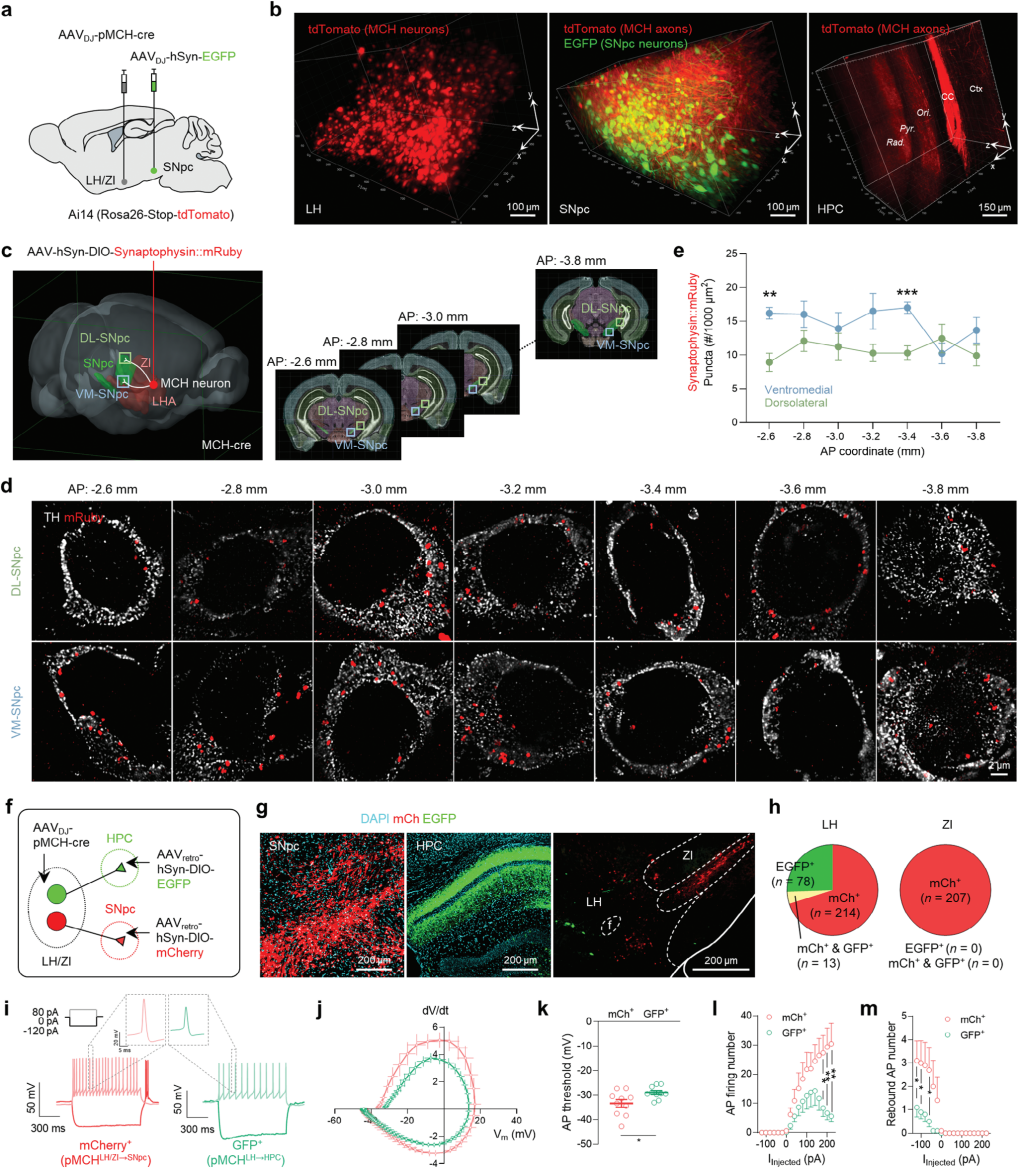
MCH^LH/ZI→SNpc^ and MCH^LH→HPC^ projections are originated from discrete neuronal subpopulations. a) Schematic diagram of viral injections for elucidating the MCH^LH/ZI^ neuronal projections. b) 3D rendering of a cleared mouse brain showing *tdTomato*‐labeled MCH^LH/ZI^ neuronal soma (left) and projections to SNpc (middle) and HPC (right). c) Schematic diagram of virus injection strategy for anatomically elucidating the MCH neuronal projections to SNpc. d) Lattice‐SIM image of synaptophysin::mRuby near the TH‐positive neurons in the SNpc. Low magnification images are displayed in Figure S5a, Supporting Information. e) Quantification of synaptophysin::mRuby‐positive dots throughout the whole SNpc. f) Schematic diagram of viral strategy for differentially labeling MCH neurons projecting to SNpc (mCherry) or HPC (EGFP). g) Representative confocal images of MCH axon fibers in SNpc or HPC originated from the soma located in LH/ZI. f, fornix. h) Intra‐LH/ZI localization and quantification of MCH neuronal subpopulations projecting to SNpc (*mCherry*; MCH^LH/ZI→SNpc^) or HPC (EGFP; MCH^LH→HPC^). i) Representative traces of membrane potentials recorded from MCH^LH/ZI→SNpc^ or MCH^LH→HPC^ neurons upon current steps (left top). j) Phase plot analyses of action potentials. k‐m) Intrinsic electrophysiological properties of MCH^LH/ZI→SNpc^ or MCH^LH→HPC^ neurons; AP threshold k), AP firing numbers upon depolarizing current step l), rebound AP numbers upon hyperpolarizing current steps (m). Statistical significance was assessed by unpaired two‐tailed Student's t‐test. ^*^ *p* < 0.05, ^**^ *p* < 0.01, ^***^ *p* < 0.001, *NS*, non‐significant. All data are presented as mean ± SEM. Detailed statistical information is listed in Table S1 (Supporting Information).

To investigate how MCH^LH/ZI^ neurons can mediate the effects of acupuncture on motor and memory deficits, we screened the projection of MCH^LH/ZI^ neurons using light‐sheet imaging with pMCH‐tdTomato mice (**Figure** **4**a,b; Figure S4a,b, Supporting Information). We observed the tdTomato^+^ axon terminals in the GFP‐labeled SNpc and HPC (Figure 4b), two regions critically involved in motor and memory deficits in PD, respectively,^[^ ^28^ ^]^ in addition to other previously known target regions (Figure S4c–e, Supporting Information).^[^ ^29^ ^]^ While the MCH^LH/ZI^ neuronal projection to HPC has been thoroughly documented,^[^ ^17^, ^19^ ^]^ its projection to SNpc^[^ ^18^ ^]^ is relatively less understood. To anatomically elucidate the MCH^LH/ZI^ neuronal projections to SNpc, we injected AAV‐DIO‐synaptophysin::mRuby virus into the LH/ZI region of MCH‐Cre mice (Figure 4c). Distinct synaptophysin::mRuby signals were discernible on TH‐positive neurons in the SNpc by adopting lattice structured illumination microscopy (Figure 4d; Figure S5a, Supporting Information). Notably, the MCH projection exhibited a significantly higher density in the ventromedial (VM) SNpc, particularly in the rostral and middle sections, compared to the dorsolateral (DL) region (Figure 4e).

Next, we sought to examine whether separate MCH^LH/ZI^ neurons project to distinct targets of the SNpc and HPC, or if a single MCH^LH/ZI^ neuronal population collateralizes onto multiple targets. To answer this question, we used an intersectional genetic approach to differentially label SNpc‐ or HPC‐projecting MCH^LH/ZI^ neurons with mCherry and GFP, respectively (Figure 4f). We found that mCherry^+^ SNpc‐projecting MCH neurons were present throughout the LH and ZI (MCH^LH/ZI→SNpc^), while GFP^+^ HPC‐projecting MCH neurons were clustered within the LH (MCH^LH→HPC^) (Figure 4g,h). Moreover, GFP^+^ and mCherry^+^ neurons were mostly separated (Figure 4h). We also observed no significant GFP^+^ fibers in the SNpc or mCherry^+^ fibers in the HPC (Figure 4g). These findings indicate that MCH^LH/ZI→SNpc^ and MCH^LH→HPC^ neurons are anatomically distinct neuronal populations that represent separate neural pathways. Subsequently, we examined the electrophysiological properties of these two subpopulations by performing *ex‐vivo* whole‐cell patch‐clamp. We found that the distinct action potential (AP) firing patterns (Figure 4i–m; Figure S5b–f, Supporting Information). MCH^LH→HPC^ neurons were characterized by lower AP firing probability (Figure 4l), while MCH^LH/ZI→SNpc^ neurons exhibited typical rebound low‐threshold bursting after hyperpolarization (Figure 4m). Taken together, our findings provided strong evidence of two discrete subpopulations of MCH^LH/ZI^ neurons: MCH^LH/ZI→SNpc^ and MCH^LH→HPC^.

To investigate whether these projections form functional circuits with target neurons in the SNpc and HPC, we performed *ex‐vivo *Ca^2+^ imaging of SNpc dopaminergic neurons and CA1 pyramidal neurons upon chemogenetic activation of MCH^LH/ZI^ neurons. We found that bath application of CNO (5 µM) was sufficient to induce a significant calcium increase in SNpc dopaminergic neurons (Figure S6a–d, Supporting Information) and CA1 pyramidal neurons (Figure S6e–h, Supporting Information). These results together indicate that distinct subpopulations of MCH^LH/ZI^ neurons send functional projections to SNpc and CA1.

### 2.5 MCH^LH/ZI→SNpc^ and MCH^LH→HPC^ Differentially Mediate Motor and Memory Function

**Figure 5:**
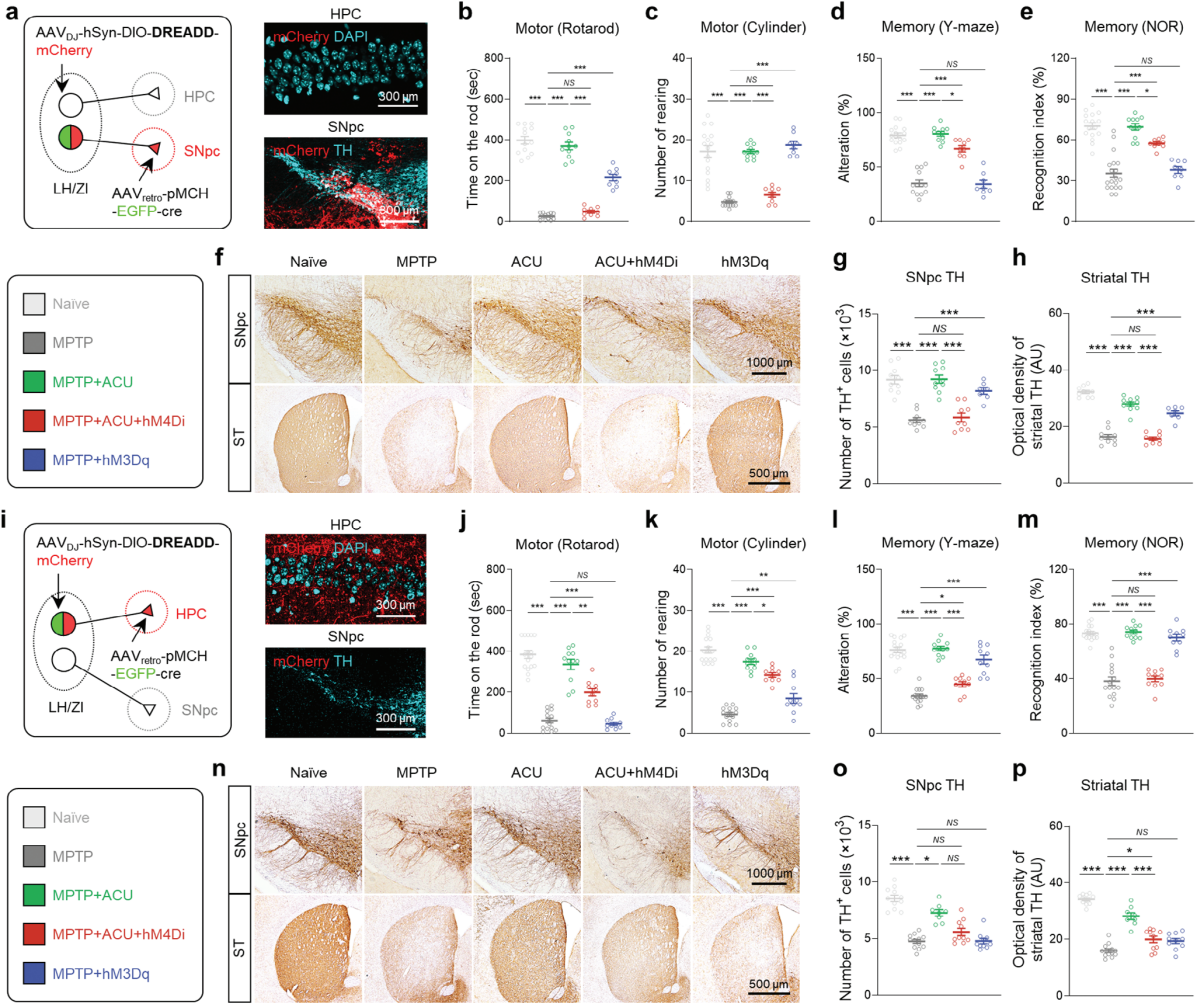
MCH^LH/ZI→SNpc^ and MCH^LH→HPC^ projections are responsible for motor and memory function, respectively. a) Schematic of viral strategy for projection‐specific chemogenetic manipulation of MCH^LH/ZI→SNpc^. b,c) Motor function assessed by rotarod tests and cylinder test. d,e) Memory function assessed by Y‐maze test and NOR test. f) Representative images of TH staining with SNpc and striatum (ST) tissues of each group. g) Numbers of TH‐positive dopaminergic neurons in the SNpc. h) Quantification of optical density of striatal TH. i) Schematic of viral strategy for projection‐specific chemogenetic manipulation of MCH^LH→HPC^. j,k) Motor function assessed by rotarod tests and cylinder test. l,m) Memory function assessed by Y‐maze test and NOR test. n) Representative images of TH staining with SNpc and striatum tissues of each group. o) Numbers of TH‐positive dopaminergic neurons in the SNpc. p) Quantification of optical density of striatal TH. Statistical significance was assessed by one‐way ANOVA with Tukey's multiple comparison test. ^*^ *p* < 0.05, ^**^ *p* < 0.01, ^***^ *p* < 0.001, *NS*, non‐significant. All data are presented as mean ± SEM. Detailed statistical information is listed in Table S1 (Supporting Information).

Since we demonstrated that MCH neurons distinctly project to the SNpc or HPC, which are the crucial brain regions of PD‐related motor and memory deficits, respectively, we next asked if the acupuncture effects can be mechanistically dissected at the circuit level. Thus, to determine whether two anatomically discrete MCH neuronal projections differentially mediate the effects on motor and memory deficits, we expressed hM4Di or hM3Dq exclusively in MCH^LH/ZI→SNpc^ or MCH^LH→HPC^ neurons in a cell type‐ and projection‐specific manner, by adopting a Cre‐dependent retrograde labeling strategy (**Figure** **5**a,i). We found that chemogenetic inhibition of MCH^LH/ZI→SNpc^ neurons significantly blocked the effects of acupuncture on motor dysfunction, with a 93.27% and 85.72% blockade in the rotarod and cylinder tests, respectively (Figure 5b,c). This chemogenetic inhibition also blocked the loss of SNpc TH‐positive cell number by 93.58% (Figure 5f,g) and completely blocked the reduction in the optical density of striatal TH (Figure 5f,h). In contrast, the effects of acupuncture on memory deficits were less affected, showing a 30.33% and 35.38% blockade in the Y‐maze and NOR tests, respectively (Figure 5d,e).

Conversely, chemogenetic activation of MCH^LH/ZI→SNpc^ neurons significantly mimicked the effects of acupuncture on motor dysfunction, with 55.27% and complete alleviation in the rotarod and cylinder tests, respectively (Figure 5b,c). This activation also alleviated the loss of SNpc TH‐positive cell number by 70.95% (Figure 5f,g) and the reduction in the optical density of striatal TH by 71.76% (Figure 5f,h). However, memory deficits were not significantly alleviated, with 0% and 7.64% alleviation in the Y‐maze and NOR tests, respectively (Figure 5d,e). These findings indicate that MCH^LH/ZI→SNpc^ neurons are responsible for acupuncture‐induced motor improvement in PD mice.

In contrast, we observed that chemogenetic inhibition of MCH^LH→HPC^ significantly abolished the effects of acupuncture on memory function, with a 75.50% and 95.33% blockade in the Y‐maze and NOR test (Figure 5l,m). On the other hand, this chemogenetic inhibition only partially affected acupuncture‐induced motor improvement, with a 49.78% and 25.26% blockade in the rotarod and cylinder tests (Figure 5j,k). Consistently, this chemogenetic inhibition also only showed a partial blockade effect on the acupuncture's impact on the loss of SNpc TH‐positive cell number and the striatal TH level (Figure 5n–p). These partial but unexpected effects of chemogenetic inhibition of MCH^LH→HPC^ neurons on motor function and nigrostriatal dopaminergic neurons could require further investigation.

Meanwhile, the chemogenetic activation of MCH^LH→HPC^ neurons significantly recapitulated the effects of acupuncture on memory function, with 76.40% and 88.33% alleviation in the Y‐maze and NOR tests, respectively (Figure 5l,m), whereas it had much less effect on the acupuncture‐mediated improvements in motor dysfunction, showing 0% and 30.49% alleviation in the rotarod and cylinder tests, respectively (Figure 5j,k). Similarly, this chemogenetic activation did not significantly rescue the nigrostriatal TH level (Figure 5n–p). Collectively, these results indicate that the two discrete MCH neuronal projections to the SNpc or HPC are differentially responsible for motor and memory recovery in the MPTP model.

### 2.6 MCH and MCHR1 are Critical for the Acupuncture Effects

**Figure 6:**
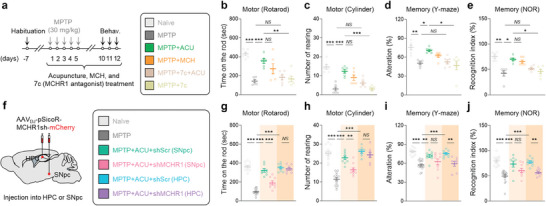
MCH‐MCHR1 activation is critical for multi‐therapeutic effects of acupuncture. a) Left, experimental timeline of various interventions including acupuncture, intranasal administration of MCH, and intraperitoneal administration of MCHR1 antagonist in the MPTP model. Right, group information. b,c) Motor function assessed by rotarod tests b) and cylinder test c) upon various interventions in the MPTP model. d,e) Memory function assessed by Y‐maze test d) and NOR test e) upon various interventions in the MPTP model. f) Schematic diagram of AAV‐mediated gene‐silencing of MCHR1. g,h) Motor function assessed by rotarod tests g) and cylinder test h). i,j) Memory function assessed by Y‐maze test i) and NOR test j). Statistical significance was assessed by Kruskal‐Wallis ANOVA test with Dunn's multiple comparison test i,j) or one‐way ANOVA with Tukey's multiple comparison test. ^*^ *p* < 0.05, ^**^ *p* < 0.01, ^***^ *p* < 0.001, *NS*, non‐significant. All data are presented as mean ± SEM. Detailed statistical information is listed in Table S1 (Supporting Information).

Since MCH neuronal activity is critical for the anti‐parkinsonian effects of acupuncture, we tested whether the neuropeptide MCH and its receptor MCHR1 are responsible for the effects. We first pharmacologically blocked MCHR1 with intraperitoneal (i.p.) administration of a selective and brain‐penetrable MCHR1 antagonist, TC‐MCH7c (10 mg kg^−1^). We found that TC‐MCH7c did not significantly affect any of the MPTP‐induced behavioral impairments or nigrostriatal TH loss (**Figure** **6**a–e). In contrast, treatment with TC‐MCH7c significantly blocked the acupuncture‐mediated alleviation of motor and memory deficits, as well as nigrostriatal TH loss (Figure 6a–e; Figure S7b,c, Supporting Information). Consistently, intranasal administration of MCH (0.5 µg in 30 µL saline) significantly recapitulated the acupuncture effects on MPTP‐induced motor deficits, TH loss, and memory impairments (Figure 6a–e; Figure S7a–c, Supporting Information).

We also tested whether the region‐specific gene‐silencing of MCHR1 by short‐hairpin RNA (shRNA) (Figure S7d–i, Supporting Information) interfered with the anti‐parkinsonian effects of acupuncture (Figure 6f–j). In line with our previous findings, MCHR1 gene‐silencing in the SNpc significantly blocked acupuncture‐mediated improvements in motor function assessed by rotarod and cylinder tests, whereas hippocampal MCHR1 gene‐silencing did not (Figure 6g,h). Likewise, MCHR1 gene‐silencing in the SNpc fully blocked acupuncture's rescuing effect in nigrostriatal dopaminergic neurons (100% blockade in SNpc TH‐positive cells; 88.76% blockade in striatal TH optical density), while hippocampal MCHR1‐shRNA resulted in significant but lesser blockade effects (63.28% blockade in SNpc TH‐positive cells; 77.10% blockade in striatal TH optical density) (Figure S7m,n, Supporting Information). Nonetheless, we observed a partial but unexpected blockade effect of hippocampal MCHR1‐shRNA treatment on the nigrostriatal TH level, which coincides with the unexpected efficacy of chemogenetic inhibition of MCH^LH→HPC^(Figure 5n–p), suggesting a hypothesis that hippocampal MCH‐MCHR1 interaction may influence nigrostriatal pathway and warranting future investigation. On the other hand, MCHR1 gene‐silencing in the HPC showed a significant blockade effect on the acupuncture‐mediated improvement of memory function in the Y‐maze and NOR tests, whereas we could not observe any significant effects with MCHR1 gene‐silencing in the SNpc (Figure 6i,j). These findings together indicate that MCHR1‐mediated MCH signaling is indeed critical for the acupuncture effect.

### 2.7 Acupuncture and MCH Neuronal Activation Commonly Alter Transcriptomic Profiling

**Figure 7:**
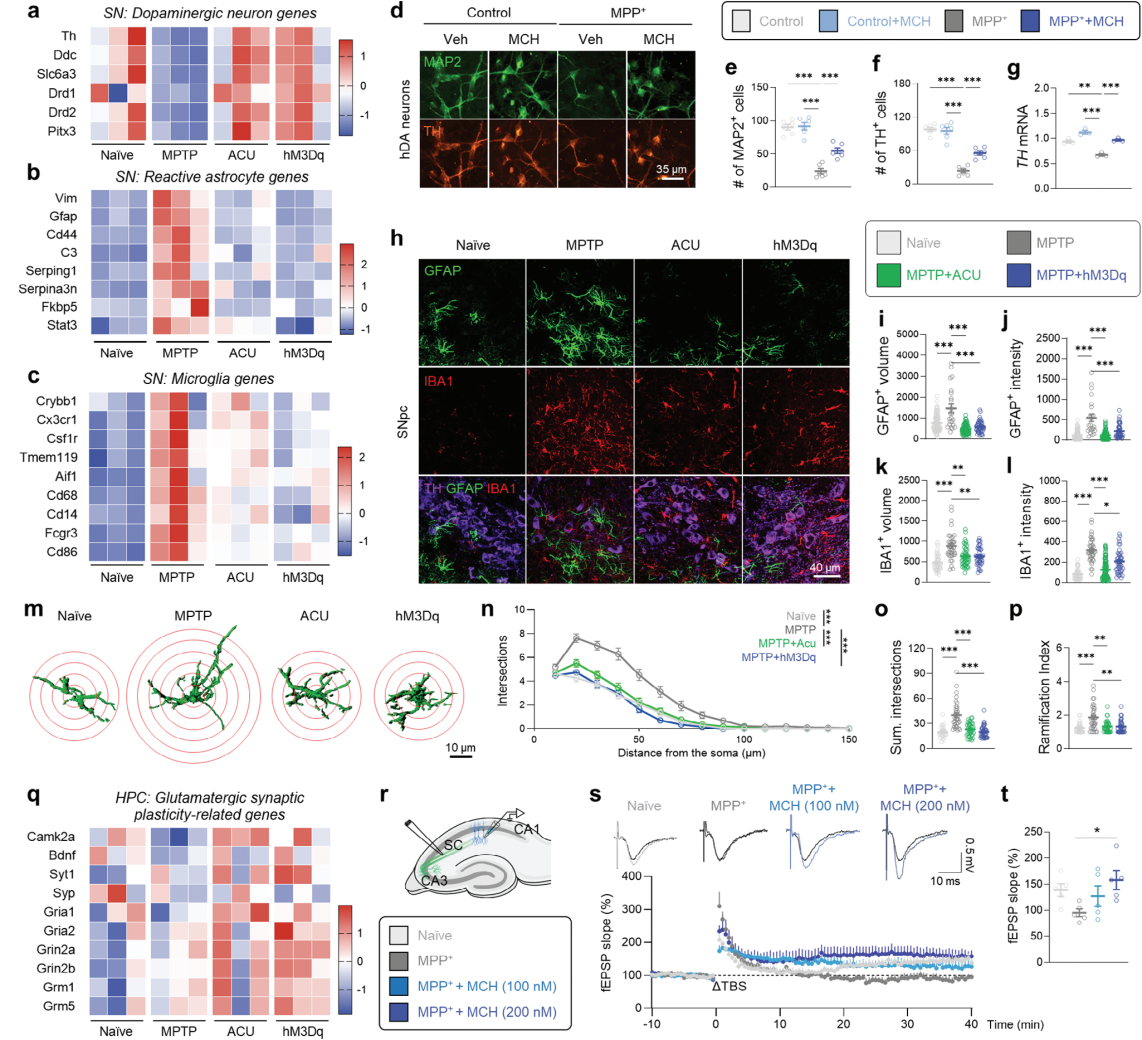
Acupuncture and MCH^LH/ZI^ activation share transcriptomic signatures. a–c) Heatmap showing enriched genes in specific cell types such as DA neurons a), reactive astrocytes b), and microglia c) in the SN. d) Immunostaining with MAP2 and TH in hDA neurons treated with MPP^+^ and MCH. e,f) The number of MAP2‐positive e) and TH‐positive cells f). g) mRNA expressions of *TH*. h) Representative confocal images of GFAP‐positive astrocyte and IBA1‐positive microglia in the SNpc. i,j) Quantification of volume i) and GFAP intensity j) of each astrocyte. k,l) Quantification of volume k) and IBA1 intensity l) of each microglia. m) Three‐dimensionally rendered images of GFAP‐positive astrocytes. Red circles indicate superimposed spheres centered around astrocyte somata used for Sholl analysis. n–p) Morphometric analyses of GFAP‐positive astrocytes by Sholl analysis; Sholl intersections by distance from the soma n), total number of intersections o), and ramification index p,q) Heatmap showing glutamatergic synaptic plasticity‐related genes in the HPC. r) Schematic diagram of *ex‐vivo* field recording for LTP measurement at SC‐CA1 synapse. s) Top, Representative fEPSP traces before (black) and 40 min after theta‐burst stimulation (TBS). Bottom, time‐course of fEPSP slope change. t) Quantification of changes in the fEPSP slopes after TBS (for the last 10 min). The expression profiles of expanded marker gene sets for dopaminergic neurons a), reactive astrocytes b), microglia c), and glutamatergic synaptic plasticity q) are shown in Figures S8f–h and S10f of the Supporting Information. Statistical significance was assessed by Kruskal‐Wallis ANOVA test with Dunn's multiple comparison test k,l,o,p), two‐way ANOVA with Tukey's multiple comparison test n), or one‐way ANOVA with Tukey's multiple comparison test. ^*^ *p* < 0.05, ^**^ *p* < 0.01, ^***^ *p* < 0.001, *NS*, non‐significant. All data are presented as mean ± SEM. Detailed statistical information is listed in Table S1 (Supporting Information).

Next, we performed RNA‐sequencing to unbiasedly explore the common mode‐of‐action of acupuncture and chemogenetic activation of MCH neurons in alleviating the PD‐related motor and memory deficits. To investigate alterations in gene expression profiles across the four groups (naïve, MPTP, MPTP+Acupuncture, and MPTP+hM3Dq), we used substantia nigra (SN) and HPC tissues from each mouse (N = 3 for each group; Figures S8a and S10a, Supporting Information). We analyzed the RNA‐sequencing data using fragments per kilobase of transcript per million mapped reads (FPKM > 1; Figure S8b–h, Supporting Information). We found that the MPTP model showed reduced expression of dopaminergic neuron genes (**Figure** **7**a; Figure S8f, Supporting Information) and increased expression of several marker genes of reactive astrocytes (Figure 7b; Figure S8g, Supporting Information) and microglia (Figure 7c; Figure S8h, Supporting Information), which were generally reversed by both acupuncture and MCH neuronal activation (Figure 7a–c; Figure S8c–h, Supporting Information). We further determined differentially expressed genes (DEGs) with a cutoff of an adjusted p‐value of less than 0.05 and a fold‐change of greater than 1.5 between the two groups. We identified 171 SN DEGs that were increased by MPTP treatment and reversed by both acupuncture and hM3Dq‐activation of MCH neurons (MPTP‐up, ACU‐down, and hM3Dq‐down) and 81 DEGs that were changed in the opposite direction (MPTP‐down, ACU‐up, and hM3Dq‐up). Notably, gene ontology (GO) analysis of these SN DEGs supported the common therapeutic effects of acupuncture and MCH neuronal activation in the MPTP model (Figure S9a–d, Supporting Information).

The increase in dopaminergic neuron‐related genes by acupuncture and MCH neuronal activation was recapitulated by direct MCH application onto human induced pluripotent stem cell (hiPSC)‐derived dopaminergic (hDA) neurons which exhibits PD‐like phenotypes by 1‐Methyl‐4‐phenylpyridinium (MPP^+^) treatment (Figure 7d–g; Figure S9e–g, Supporting Information). This result suggests that MCH can elicit neuroprotective action in a cell‐autonomous manner. On the other hand, the reduction in gliosis‐related transcriptomic profiling in the SN tissue, particularly astrogliosis, is also noteworthy because previous reports have demonstrated that reactive astrocytes can lead to dopaminergic neurodegeneration in a non‐cell‐autonomous manner in PD pathology.^[^ ^26^, ^30^ ^]^ Our immunohistochemistry results demonstrated that the intensities of GFAP and IBA1, as well as cellular volumes of astrocytes and microglia, were significantly increased in the SNpc of the MPTP model, which were reversed by both acupuncture and chemogenetic activation of MCH neurons (Figure 7h–l). Additionally, the high ramification of astrocytes, a key morphological characteristic of reactive astrocytes, was significantly reduced by both acupuncture and chemogenetic activation of MCH neurons (Figure 7m–p). These findings indicate that acupuncture and the associated MCH neuronal activation can alleviate the MPTP‐induced reactive gliosis. Taken together, these findings suggest that MCH can restore the dopaminergic neurons via both non‐cell‐autonomous and cell‐autonomous mechanisms, which is responsible for the anti‐parkinsonian effect of acupuncture.

In the HPC, we observed a reduction in the expression of genes related to glutamatergic synaptic plasticity and homeostatic microglial action, whereas there was an increasing trend in the expression of genes related to reactive astrocytes and pro‐inflammatory microglia following MPTP treatment. Acupuncture and hM3Dq‐mediated MCH neuronal activation alleviated these changes (Figure 7q; Figure S10, Supporting Information). Particularly, GO analysis with 27 HPC DEGs (MPTP‐up, ACU‐down, and hM3Dq‐down) demonstrated that inflammatory signaling was attenuated by acupuncture and MCH neuronal activation (Figure S11, Supporting Information). These findings indicate that acupuncture and chemogenetic activation of MCH neurons commonly alter the transcriptomic profiling of the SN and HPC of the MPTP model toward the normal state. Interestingly, we revealed that MCH was sufficient to recapitulate the effects of acupuncture and hM3Dq‐mediated MCH neuronal activation in restoring the hippocampal synaptic plasticity (Figure 7r–t). These findings strongly suggest that MCH is the essential molecular substance for the anti‐parkinsonian effects of acupuncture in the brain. Altogether, our findings strongly suggest that the anti‐parkinsonian effects of acupuncture are attributed to MCH‐mediated alleviation of reactive gliosis, protection of dopaminergic neurons in the SNpc, and enhancement of glutamatergic synaptic plasticity in the HPC.

## 3 Discussion

The neurobiological mechanisms underlying how acupuncture stimulation at a peripheral acupoint modulates neural activity in the brain has remained unclear for several millennia. Our study demonstrated that acupuncture stimulation at a hindlimb acupoint GB34 activates MCH^LH/ZI^ neurons via afferent nerve conduction. Through cell type‐ and projection‐specific chemogenetic manipulation with an intersectional genetic approach, we demonstrated that acupuncture can simultaneously alleviate the PD‐related motor and memory deficits through MCH neuronal activation. We further identified two subpopulations of MCH^LH/ZI^ neurons and delineated their discrete functional relevance: motor behaviors (MCH^LH/ZI→SNpc^) and non‐motor behaviors (MCH^LH→HPC^). This finding is noteworthy because it has not been studied whether the separate subpopulations of MCH^LH/ZI^ neurons have distinct projections involving differential functions in the system level, despite recent highlights on the diversity of several hypothalamic neurons.^[^ ^31^ ^]^ Our findings strongly suggest that these two distinct circuits from MCH^LH/ZI^ neurons are the cellular and circuit‐level neuroanatomical basis of the multi‐function of acupuncture in PD (Figure S12, Supporting Information).

PD pathophysiology primarily involves the progressive degeneration of dopaminergic neurons in the SNpc, resulting in motor impairments such as bradykinesia, rigidity, and resting tremor. Additionally, it is widely recognized that the majority of patients eventually develop various cognitive impairments, such as memory loss, which are markedly dependent on HPC dysfunction. Indeed, HPC atrophy has been well documented in PD patients.^[^ ^32^ ^]^ Beyond the SNpc and HPC, other brain areas, such as the LH, are also affected in PD patients, as evidenced by a loss of MCH neurons and orexinergic neurons.^[^ ^15a^ ^]^ While its pathological role and therapeutic value have been underestimated, one previous report has demonstrated that LH has a potential for controlling the motor function by regulating SNpc.^[^ ^33^ ^]^ ZI is also well known for its potential in regulating motor function, especially when targeted with DBS in PD patients.^[^ ^16^ ^]^ In addition to motor function, the LH is well documented for its physiological role in memory function through LH→HPC circuit.^[^ ^17b,c^ ^]^ Altogether, these previous findings suggest that the LH/ZI, as the controlling center of both motor and memory function, could be revisited as a therapeutic target for PD.

It is worth noting that MCH^LH/ZI^ neurons have at least two anatomically, electrophysiologically, and functionally distinct subpopulations that project their axons to different target regions: MCH^LH/ZI→SNpc^ and MCH^LH→HPC^. While the diversity of several hypothalamic neurons has long been accentuated,^[^ ^31^, ^34^ ^]^ MCH^LH/ZI^ neurons have been reported to be categorized into two or more subpopulations based on their molecular and electrophysiological properties.^[^ ^31^, ^35^ ^]^ However, it has been unclear whether the separate subpopulations have distinct projections which mediate differential functions in the neural circuit level. Our study demonstrated that MCH neuronal projections to the SNpc and HPC originated from two distinct subpopulations that differ anatomically and electrophysiologically. Moreover, we found that the chemogenetic activation of the two MCH^LH/ZI^ neuronal subpopulations led to completely different therapeutic effects in the PD mouse model: alleviating the motor and memory deficits, respectively. Our findings provided concrete evidence that the differential effects of two MCH subpopulations were mainly attributed to their discrete target regions.

In previous studies, we and others have provided accumulated lines of evidence on the acupuncture's anti‐parkinsonian effects. In detail, these studies demonstrated that acupuncture at GB34 can reinforce dopamine availability,^[^ ^8^ ^]^ increase neuroprotective molecules,^[^ ^9a,c^ ^]^ and alleviate oxidative stress and inflammation ^[^ ^9^, ^10^ ^]^ in PD mouse models.^[^ ^11^ ^]^ Nevertheless, a significant gap has remained between peripheral acupuncture stimulation and the molecular changes in the brain. Our study bridges the gap by demonstrating that peripheral acupuncture stimulation activates MCH^LH/ZI^ neurons via afferent nerve conduction and that MCH released from two distinct MCH^LH/ZI^ neuronal projections (i.e., MCH^LH→HPC^ and MCH^LH/ZI→SNpc^) is critical for the molecular changes observed in the SNpc and HPC. Our current study introduces a novel approach to understanding the circuit‐level mechanisms underlying acupuncture's effects in PD, providing insights into how acupuncture can simultaneously alleviate both motor and non‐motor symptoms of the disease.

We have revealed that the anti‐parkinsonian effects of acupuncture in both PD‐related motor and non‐motor symptoms were attributed to the action of MCH. First, while the motor symptoms of PD are known to result from the extensive nigrostriatal dopaminergic neuronal death, the non‐cell‐autonomous mechanisms through reactive astrocytes and microglia have also been reported.^[^ ^26^, ^30^, ^36^ ^]^ Our study demonstrates that MCH can protect dopaminergic neurons both cell‐autonomously and non‐cell‐autonomously via reducing reactive gliosis. Second, while the cardinal motor symptoms have been emphasized as the primary target for most existing PD treatments, the non‐motor symptoms of PD, including cognitive impairment, have received little attention, despite their clinical importance. This study demonstrates that acupuncture treatment significantly recovers the impairments in LTP at the Schaffer collateral‐CA1 synapse in the HPC and the spatial memory impairment in PD mice, through the MCH‐MCHR1 interaction. This is in line with previous findings showing that MCHR1 mRNA is highly expressed in the CA1 HPC^[^ ^37^ ^]^ and that MCH might contribute to hippocampal synaptic plasticity and memory behaviors through the signaling pathway involving BDNF and its receptor, tropomyosin receptor kinase B (TrkB).^[^ ^38^ ^]^ Taken together, our results propose MCH as the potential therapeutic target as well as molecular basis of anti‐parkinsonian effects of acupuncture on both motor and non‐motor symptoms.

In this study, we revealed the precise cellular and circuit‐level mechanisms of anti‐parkinsonian effects of acupuncture via MCH^LH/ZI^ neurons. However, our study lacked investigation into the peripheral mechanisms that govern how mechanical stimulation by acupuncture needling initiates the entire therapeutic effects in the skin and muscular layers. Based on a recent study demonstrating that the activity of PROKR2‐positive sensory neurons is critical for the anti‐inflammatory effects of acupuncture,^[^ ^4^ ^]^ it would be interesting to study whether this specific subset of sensory neurons is also crucial for anti‐parkinsonian effects.

This study demonstrates that peripheral acupuncture stimulation can regulate specific central neural pathways, particularly those involving MCH^LH/ZI^ neurons, affecting multiple brain regions such as SNpc and HPC. This regulation potentially improves both motor and non‐motor symptoms of PD. Unlike invasive procedures such as DBS, acupuncture is a relatively safe treatment with minimal side effects,^[^ ^39^ ^]^ making it suitable for long‐term use in PD patients. Our previous findings also showed that acupuncture could reduce LID and enhance levodopa's effects,^[^ ^13^, ^40^ ^]^ suggesting that acupuncture could be integrated with existing PD therapies for sustained management. Overall, acupuncture appears to be a promising therapeutic approach for managing PD, offering the ability to target both motor and non‐motor symptoms through a less invasive means. Further research is needed to fully understand its mechanisms and broader clinical applications in PD management.

## 4 Conclusion

Current PD therapies have only focused on symptomatic relief through dopamine supplementation or dopamine degradation blockade, which cannot modify the disease progression. However, our findings suggest that MCH may have the potential to modify the disease progression of PD through its ability to promote dopaminergic neuroprotection and reduce reactive gliosis. We further propose peripheral nerve stimulation by acupuncture could be an effective strategy for eliciting MCH^LH/ZI^ neuronal activity to relieve motor and non‐motor symptoms in PD. Taken together, our study proposes the neuroanatomical basis of the anti‐parkinsonian effects of acupuncture, which was previously considered a non‐scientific intervention. These findings shed light on an exciting new avenue for PD therapy.

## 5 Experimental Section

### Mice

All animal experimental procedures were conducted in accordance with the National Institutes of Health guidelines and approved by the Institutional Animal Ethical Committee, Kyung Hee University (Seoul, Korea, Approval Number KHSASP‐19‐412), and Korea Institute of Science and Technology (KIST; Seoul, Korea, Approval Numbers KIST‐2017‐026 and KIST‐2021‐097). If there was no specific explanation, 8–10‐week‐old male *C57BL/6J* mice were used for experiments. *Ai148* (B6.Cg‐*Igs7^148.1(tetO‐GCaMP6f,CAG‐tTA2)Hze^*/J) transgenic mice were used for in‐vivo and ex‐vivo Ca^2+^ imaging. *CamK2a‐Cre* transgenic mice crossed with *Ai148* transgenic mice were used for ex‐vivo Ca^2+^ imaging of pyramidal neurons in the CA1 HPC. *Ai14* (B6;129S6‐*Gt(ROSA)^26Sor14(CAG‐tdTomato)Hze^* ^/^J) transgenic reporter mice were used for visualizing the MCH neurons and their projections with *AAV_DJ_‐pMCH‐Cre* virus injection. MCH‐Cre transgenic mouse (#01 4099, Jax, USA) was used to investigate MCH projection from the LH/ZI to the SNpc with *AAV_5_‐hSyn‐DIO‐synaptophysin‐mRuby* virus injection into the LH/ZI. All mice were co‐housed at the animal facility with a temperature of 21 ± 2 °C, a relative humidity of 50 ± 15%, and a 12 h light/dark cycle (lights on and off at 8 am and 8 pm). All mice were provided with food and water ad libitum. All mice were randomly allocated to each experimental group. All experiments were performed with age‐matched controls and different sets of animals were used for each experiment.

### Virus Preparation and Stereotaxic Injection

For all surgical procedures, mice were anesthetized with 2% isoflurane and head‐fixed in a mouse stereotaxic apparatus (#68 513, RWD, USA; Model 940, KOPF, USA). All virus injections were delivered at a rate of 0.2 µL min^−1^ via a blunt‐end injection needle (33‐gauge, CMA, USA) using a microinjection syringe pump (KDS 310, KD Scientific, USA).

To visualize the MCH neurons or chemogenetically manipulate their activity, AAV_DJ_‐pMCH‐EGFP‐Cre, AAV_DJ_‐pMCH‐Cre, AAV_DJ_‐hSyn‐DIO‐hM3Dq‐mCherry, or AAV_DJ_‐hSyn‐DIO‐hM4Di‐mCherry into the LH (AP = −1.0 mm, ML = ±1.2 mm from bregma, DV = −4.85 mm from dura) was injected. The viruses injected into the LH were repeatedly spread to the ZI. To visualize the MCH^LH→HPC^ or MCH^LH/ZI→SNpc^ projection or retrogradely express Cre in MCH^LH→HPC^ or MCH^LH/ZI→SNpc^ neurons, AAV_retro_‐hSyn‐DIO‐EGFP, AAV_retro_‐hSyn‐DIO‐mCherry or AAV_retro_‐pMCH‐EGFP‐Cre into the CA1 HPC (AP = −1.94 mm, ML = ±1.5 mm from bregma, DV = −1.3 mm from dura) or SNpc (AP = −3.2 mm, ML = ±1.25 mm from bregma, DV = −4.0 mm from dura) was injected. To investigate MCH projection from the LH/ZI to the SNpc, AAV5‐hSyn‐DIO‐synaptophysin‐mRuby into the LH (AP = −1.0 mm, ML = ±1.2 mm from bregma, DV = −4.85 mm from dura) was injected. For ex‐vivo Ca^2+^ imaging of dopaminergic neurons in the SNpc, AAV_DJ_‐DDC‐Cre was injected into the SNpc of *Ai148* transgenic mice. To generate the viral *A53T* overexpression‐induced PD mouse model,^[^ ^26^ ^]^ AAV_DJ_‐CMV‐A53T‐SNCA into the SNpc. For gene‐silencing of MCHR1 expression in HPC and SNpc was injected, AAV_DJ_‐pSicoR‐MCHR1sh‐mCherry into CA1 HPC or SNpc was injected. All AAVs were manufactured by the KIST Research Animal and Resource Center (KIST RARC). For viral trans‐synaptic tracing from GB34 to the dorsal root ganglion (DRG), spinal cord, and brain, PRV‐CMV‐EGFP (P01001, BrainVTA, China) and PRV‐CMV‐RFP (P01002, BrainVTA) were utilized. 1.5 uL of PRV‐CMV‐EGFP into the LH/ZI region was injected. To examine DRGs containing neurons both projected from the peripheral acupoint and projecting to the brain, PRV‐CMV‐RFP was injected into the GB34 with three distinct depths: 2 mm (muscular layer), 1.5, and 1 mm (subcutaneous layer). The detailed information about virus titers, volumes, and total amount for each experiment is listed in Table S2 (Supporting Information).

### MPTP‐Induced Parkinsonian Model in Mice

All MPTP experiments used the subchronic regimen consisting of a daily i.p. injection of MPTP (30 mg kg^−1^ per day, 23007‐85‐4, Sigma–Aldrich, USA) for five consecutive days. Control animals received saline injections only.

### Acupuncture Treatment

Acupuncture treatment was performed 2 h after MPTP injection for 12 consecutive days by inserting a stainless‐steel acupuncture needle (15 mm in length, 0.20 mm diameter, Haeng‐lim‐seo‐weon Acuneedle Co, Korea) with a depth of ≈3 mm bilaterally at the GB34 acupoint. The GB34 acupoint, located at the intersection point of lines from the anterior borders to the head of the fibula, had been used to treat movement disorders in traditional East Asian medicine, and recent neuroimaging studies have confirmed its effect on motor function.^[^ ^6^, ^7^, ^41^ ^]^ Acupuncture stimulation at GB34 acupoint had been reported to be effective for alleviating motor symptoms in PD animal models.^[^ ^8^, ^9^, ^42^ ^]^ A control non‐acupoint (nonACU) located at the point ≈3 mm lateral side of a tail on the gluteus muscle was chosen. These inserted needles were then turned at a rate of two spins per sec for 30 s and removed immediately. Treatment was performed accurately and quickly to minimize stress on the mice. Also, mice in all groups were mildly immobilized by holding their necks, with the head in an upright position for 30 s to give the same immobilization stress as the acupuncture group.

### Measurements of the Displacement and Rotational Frequency

To standardize and stabilize acupuncture procedures before treatment, Acusensor (Stromatec, Inc., Burlington, VT, United States) was used by a trained practitioner. The Acusensor detects the two main components of needle motion during manual manipulation: “displacement” and “rotation frequency”.^[^ ^43^ ^]^ Both displacement and rotation frequency were measured by an optical sensor that visualizes the movement of microscopic asperities on the needle's surface. This sensor provides measurements with a resolution of up to 2000 counts per inch of needle motion, at a frame rate of up to 7 kHz. The sensor and its associated electronics were housed in a small module measuring ≈30 × 20 and 10 mm thick. A sterile, single‐use, disposable needle guide with a 350 µm diameter hole allows the acupuncture needle to pass through, ensuring the optical sensor was focused on the needle's shaft to detect its rotation and displacement. An onboard microprocessor continually sums the incremental measurements from the optical sensor to produce absolute position estimates. During use, the practitioner identifies the point to be needled and places the motion sensor over this point. A sterile disposable acupuncture needle was then inserted through the needle guide into the acupoint on a mouse. The practitioner holds the motion sensor against the mouse's acupoint with their free hand while performing the needling. Before use, the motion sensors were calibrated using a computer‐controlled two‐axis actuator that generates known needle motions. These known motions were compared to the raw motion measurements to compute calibration coefficients in the form of scale factors and offsets.

### Sciatic Axotomy and Lidocaine Treatment

For sciatic nerve transection, mice were anesthetized with rompun (100 µL, i.p.; Bayer, Korea) and 2% zoletil (150 µL, i.p.; Virbac S.A, France). The bilateral hind thigh was shaved and received a skin incision at mid‐thigh level and the sciatic nerve was exposed. Then, the nerves were transected, and a ligature was performed in the proximal segment in order to prevent spontaneous reinnervation. For blocking the axonal reflex with a local anesthetic, 10 µL lidocaine (lidocaine‐HCl 400 mg; Huons, Korea) was intradermally injected at the acupoint 5 min before acupuncture stimulation.

### Chemogenetic Manipulation

To manipulate the activity of MCH neurons in LH/ZI, we adopted *Cre*‐dependent DREADD expression and i.p. administration of CNO. Specifically, *AAV_DJ_‐pMCH‐Cre* virus into LH/ZI to express *Cre* specifically in MCH neurons was injected. Simultaneously, *AAV_DJ_‐hSyn‐DIO‐hM4Di‐mCherry* or *AAV_DJ_‐hSyn‐DIO‐hM3Dq‐mCherry* virus into LH/ZI to Cre‐dependently express Gi‐DREADD or Gq‐DREADD in the *Cre*‐induced MCH^LH/ZI^ neuron was injected, respectively. Two weeks later, it was started to administer MPTP. To activate Gi‐DREADD and Gq‐DREADD, it was intraperitoneally administered CNO at a dose of 1 mg kg^−1^ for 12 days. In terms of Gi‐DREADD‐mediated inhibition, we treated with CNO 30 min before acupuncture stimulation. For circuit‐specific manipulation, *AAV_retro_‐hSyn‐DIO‐hM4Di‐mCherry* and *AAV_retro_‐hSyn‐DIO‐hM3Dq‐mCherry* virus into HPC or SNpc was injected.

For chemogenetic activation of sensory afferents at GB34 acupoint, we injected *AAV_retro_‐hSyn‐hM3Dq‐mCherry* into bilateral GB34 at the three different depths of 1, 1.5, and 2 mm for targeting subcutaneous, superficial, and deep muscular layers to mimic the acupuncture effects by chemogenetic activation of afferent nerve fibers surrounding the GB34 acupoint. Two weeks later, CNO (1 mg kg^−1^, 50 uL in total) into the bilateral GB34 was injected.

### Administration of MCH and MCHR1 Blocker

To assess the possible therapeutic effects of MCH in the MPTP mouse model, mice were intranasally administered with MCH (0.5 µg in 30 µL saline; #3806, Tocris, UK) for 12 consecutive days 2 h after MPTP treatment. To pharmacologically inhibit MCHR1, 10 mg of MCHR1 antagonist TC‐MCH7c (7c; 10 mg kg^−1^, dissolved in 8 mL of 1% dimethyl sulfoxide) was intraperitoneally administered to the mice 30 min before the acupuncture treatment.

### Motor Behavioral Assays

*Rotarod test*. An accelerated rotarod test was conducted to evaluate the coordination and balance of motor function.^[^ ^44^ ^]^ On the day of testing, mice were kept in their home cages and acclimated to the testing room for at least 30 min. A 2 min acclimation session at 2 rpm was performed on the first day before the test phase. Rotarod testing (MED Associates, Inc, USA) involves placing mice on a rotating bar and determining the length of time that they can retain their balance while the rotation speed increases over 480 s. The speed is increased from 3.5 to 35 rpm, reaching maximum speed at 5 min. Each trial was terminated when the mouse fell off. The latency to fall from the rotating rod was scored by automatic timers and falling sensors on the rotarod.

*Cylinder test*. A cylinder test was performed to evaluate the spontaneous explorative behavior in a new environment.^[^ ^45^ ^]^ Mice were placed in a transparent plastic cylinder (12 cm in diameter × 20 cm tall) for 1 min before the experiment. After adaptation, when mice tried to explore different areas of the cylinder by standing on their hindlimbs and leaning with the forelimbs on the cylinder wall, the cylinder wall touches (numbers) were counted by observers for 3 min.

*Adhesive removal test*. The adhesive removal test was established in alpha‐synuclein *A53T* mice to determine the effects of MCH activation on sensory‐motor behavior.^[^ ^46^ ^]^ Mice were placed in a clean cage and allowed to habituate for 30 min. A small circular adhesive paper sticker (5 mm in diameter) was gently but firmly attached to the snout. The ability to remove the adhesive sticker was evaluated by the time required for mice to remove the sticker. Mice were given 60 s to complete this sensorimotor task. Each mouse was subjected to three trials separated by 1 min rest and the results were averaged.

### Memory Behavioral Assays

*Y‐maze test*. Spatial working memory was measured by spontaneous alternation behavior in the Y‐maze.^[^ ^47^ ^]^ The Y‐maze consisted of three arms (4 × 30 × 15 cm) that were the same, with a 120° angle between each of the two arms. Each mouse was placed in one of the Y‐maze arms and allowed to explore the maze freely for 5 min. The sequence and total number of arms entered were recorded. An arm entry was considered complete when both hind paws were in the arm. The apparatus was cleaned with water and ethanol between each passage. The percentage of spontaneous alternation was determined by the number of trials containing entries into all three arms/maximum possible alternations (total number of arms entered – 2) × 100.

*Novel object recognition test*. Short‐term memory was evaluated by performing a novel object recognition test.^[^ ^48^ ^]^ The apparatus consisted of a 60 × 60 × 30 cm acrylic box with white walls and a floor. Animals received 5‐min sessions in the empty box for habituation to the apparatus and test room. Then, 24 h later, each mouse was exposed to two familiar objects (round block, 4 cm in diameter) during a 5‐min training stage in the box. Next, the animals were placed back in the box and exposed to novel object (rectangle block, 4 × 4 × 4 cm) and the familiar object for another 5 min (test stage), 24 h after the training stage. The time spent exploring each object was measured. The recognition index reflecting the short‐term memory ability was calculated as the ratio of time spent exploring the novel object over the total exploration time. It was excluded data where the total exploration time for both objects was less than 20 s, as it was deemed insufficient for adequate learning or discrimination exploration.^[^ ^49^ ^]^

### Immunohistochemistry, Confocal Microscopy, and Slide Scanner Imaging

For all histological analyses, mice were deeply anesthetized and transcardially perfused with phosphate‐buffered saline (PBS) followed by 4% paraformaldehyde (PFA) in 0.2 M phosphate buffer. The brains and hindlimbs were removed, post‐fixed in 4% PFA, and cryoprotected in 30% sucrose at 4 °C for three days. Brains and hindlimbs were divided into 30‐µm thick coronal sections on a cryostat microtome (Leica Biosystems, Germany). The tissue sections were first incubated for 1.5 h in a blocking solution (0.3% Triton‐X, 2% goat serum, and 2% donkey serum in 0.1 M PBS) and then immunostained with a mixture of primary antibodies in a blocking solution at 4 °C. Primary antibodies used were as follow: rabbit anti‐TH (1:1000; sc‐14007, Santa Cruz, USA), rabbit anti‐c‐Fos (1:500; sc‐253, Santa Cruz), goat anti‐pMCH (1:500; sc‐14507, Santa Cruz), mouse anti‐NeuN (1:1000; MAB377, Millipore), goat anti‐MCH1R (1:500; sc‐5534, Santa Cruz), goat anti‐Iba1 (1:500; ab5076, Abcam), and chicken anti‐NEFH (1:500, ab4680, abcam). After washing three times in PBS, the sections were incubated in the corresponding fluorescent secondary antibodies for 1 h at room temperature. Then, they were washed with PBS three times. If needed, DAPI (1:3000, Pierce) staining was performed. Finally, sections were mounted with a fluorescent mounting medium (S3023, Agilent, USA) and dried. A series of fluorescent images were obtained with an A1 Nikon or FV‐1000 Olympus confocal microscopes, and Z‐stack images in 3‐µm steps were processed for further analysis using NIS‐Elements (Nikon, Japan) software and ImageJ program (NIH, MD, USA). Any alterations in brightness or contrast were equally applied to the entire image set. Specificity of primary antibody and immunoreaction was confirmed by omitting primary antibodies or changing fluorescent probes of the secondary antibodies.

For confirmation of PRV‐EGFP infection in the LH, samples were scanned with a slide scanner (Axio Scan. Z1, ZEISS, Germany) and 20x images were obtained and tiled.

### Lattice Structured Illumination Microscopy (Lattice‐SIM)

2 weeks after viral infection of synaptophysin in the MCH^LH/ZI^ neuron of MCH‐Cre mice, the mice were transcardially perfused with PBS and 4% PFA. Then, 30‐µm SNpc brain slices were prepared from AP −2.6 to −3.8 mm (from bregma). Immunohistochemistry was performed with rabbit anti‐TH (1:1000; sc‐14007, Santa Cruz, USA) and DAPI (1:3000, Pierce).

The Lattice Structured Illumination Microscopy (Lattice‐SIM) technique was used to obtain wide field‐based super‐resolution images. Sample images were collected using an Elyra 7 Axio Observer inverted stand (Carl Zeiss), equipped with a Plan Apochromat 63×/1.4 oil DIC M27 objective lens, a sCMOS camera (PCO. Edge 4.2) and 4 illumination lasers (405/488/56/642). The laser lines used were 488 nm (500 mW) and 561 nm (500 mW), with a total output power of 2.3% and 2.8%, respectively. The raw images were composed of 13 phase images per plane per channel, with grating grids (27.5 and 32 um grids for the 488 and 561 laser lines respectively) automatically chosen to provide optimal resolution enhancement for both laser lines and modulation contrast. All images were taken with an exposure time of 50 ms. The processed images were obtained using Zen (black edition) software with SIM2 module (fixed standard option selected) settings. The final image size was 2048 × 2048 pixels with a size of 82.54 um × 82.54 um.

To investigate the spatio‐specific MCH neuronal projections to the SNpc, we divided SNpc into two subregions, DL and VM SNpc and obtained two images from each subregion of every SNpc slice. The mRuby‐positive spots were counted using Spot module of Imaris 9.0 (Oxford Instruments).

### DAB Staining and Stereological Quantification

The 30‐µm thick coronal sections for SNpc and striatum were immunostained with a DAB peroxidase substrate kit (SK‐4100, Vector Laboratories Inc, USA). The sections were blocked in a solution comprised of 0.3% BSA and 3% Triton X‐100 for 1 h. Then samples were activated by using anti‐TH primary antibody (1:3000, sc‐14007, Santa Cruz). Thereafter, sections were incubated with biotinylated secondary antibody (BA‐1000, Vector Laboratories Inc., USA) for 1 h. After incubation, sections were activated by avidin‐biotinylated peroxidase complex (Vectastain Elite ABC kit, Vector Laboratories Inc.) solution for 1.5 h. Sections were then developed with DAB peroxidase substrate kit. Finally, sections were mounted with mounting solution and dried. A series of bright field images of TH‐positive cells and fibers in the SNpc and striatum were obtained by using a microscope (BX53, Olympus, Japan).

An unbiased 3D counting method, stereological estimation of the total number of TH‐positive neurons in the SNpc area was performed using the optical fractionator method using Stereo Investigator 11 (11.01.2 64‐bit, MBF Bioscience, USA). For stereological purposes, unilateral sets of 1/6 section, ≈8 systematically random sections, of ≈240 µm apart, were taken from the brain spanning in all mice. The counted sections covered the rostral tip of the SNpc. An unbiased counting frame of known area (48 × 36 µm = 1728 µm^2^) was placed randomly on the first counting area and systematically moved through all counting areas (166.2 × 111.12 µm = 18468 µm^2^) until the entire delineated area was sampled. Counting was performed using a low magnification objective lens (×10). The estimated total number of positive neurons was calculated according to the optical fractionator formula.

### In‐Vivo Microendoscopy

*Imaging*. A microendoscope (nVista 2.0, Inscopix, USA) was used to record calcium signals from MCH neurons within LH in mice. To observe calcium activities of MCH cells, *Ai148* transgenic mice were injected with 1.5 µL of AAV‐pMCH‐Cre virus diluted by 1:5 (resulting titer: 1.14×10^13 GC/mL) into the left LH. Two weeks after virus injection, mice were implanted with GRIN lenses (ProView Integrated Lens – 0.5 mm diameter, 8.4 mm length, Inscopix, USA) with attached magnetic bases able to fix the microendoscope. Lens tips were fixed in LH (AP: −1.0 mm, ML: −1.2 mm from bregma, DV: −4.8 mm from skull surface). After confirming that mice showed calcium activity, mice were anesthetized by 3% isoflurane, and then maintained at 1% isoflurane for the light anesthesia during the experiment session. A typical session involved ≈10 min of calcium signal acquisition using Inscopix Data Acquisition Software at 20 frames per second and simultaneous acupuncture stimuli, both with and without lidocaine administration. Excitation laser power and digital gain were customized for each mouse according to the conditions of its cell population.

*Processing*. Raw data obtained were processed with Mosaic data processing software (Inscopix Data Processing 1.2.0, Inscopix, USA), and, first underwent motion correction to control potential motion artifacts. Next, the software normalized the brightness every pixel value in the raw video, thus generating each pixel's deviation trace, dF/F_0_, from the mean baseline. An inbuilt Principle Component Analysis and Independent Component Analysis algorithm automatically generated cell candidates based on pixels exhibiting synchronized spatial and temporal brightness changes. The resulting regions of interest representing candidate neurons were further filtered manually based on cell shape and signal‐to‐noise ratio. Resulting cell calcium activity traces were aligned with the experiment footage.

### SHIELD Tissue Processing and Light‐Sheet Microscopy

Mice were transcardially perfused with ice‐cold PBS and then with the SHIELD perfusion solution (PCK‐500, Passive clearing kit, Lifecanvas, USA) as described previously.^[^ ^50^ ^]^ Dissected brains were incubated in the same perfusion solution at 4 °C for 48 h. Tissues were then transferred to the SHIELD‐OFF solution and incubated at 4 °C for 24 h. Following the SHIELD‐OFF step, brains were placed in the SHIELD‐ON solution and incubated at 37 °C for 24 h. SHIELD‐fixed brains were cleared passively for a couple of weeks at 45 °C in a buffer solution. For optical clearing, delipidated tissues were incubated in a solution of 50% EasyIndex diluted in PBS overnight at 37 °C. The solution was then changed to 100% EasyIndex and incubated overnight at 37 °C. Tissues became transparent without any visible haze at the tissue–medium interface. 3D light‐sheet images were taken by Zeiss Light sheet Fluorescence microscopy (LSFM) 7.

### Slice Electrophysiology

*Acute brain slice preparation*. Mice were anesthetized using vaporized isoflurane. Brain was quickly removed and immersed in an ice‐cold cutting solution that contained (in mM): 130 NaCl, 24 NaHCO_3_, 3.5 KCl, 1.25 NaH_2_PO_4_, 3.0 MgCl_2_, 1.0 CaCl_2_ and 10 d‐glucose, pH 7.4. All the solution was gassed with 95% O_2_ and 5% CO_2_. After trimming the hemisected cerebrum, 300‐µm‐thick horizontal slices were cut using vibrating microtome (Neo LinearSlicer MT, DSK, Japan) and transferred to an artificial cerebrospinal fluid (aCSF) recording solution (in mM): 130 NaCl, 24 NaHCO_3_, 3.5 KCl, 1.25 NaH_2_PO_4_, 1.5 MgCl_2_, 1.5 CaCl_2_ and 10 d‐(+)‐ glucose, pH 7.4. Slices were incubated at room temperature for 1 h before recording.

*Action potential recording*. Slices were transferred to a recording chamber that was perfused with aSCF solution. The slice chamber was mounted on the stage of an upright Olympus microscope and viewed with a 60x objective lens. Cellular morphology was visualized by charge‐coupled device camera and the Imaging Workbench software (INDEC BioSystems). GFP^+^ or mCherry^+^ MCH^LH/ZI^ neurons (MCH^LH→HPC^ and MCH^LH/ZI→SNpc^ neurons, respectively) were patched with whole‐cell configuration. The holding potential was −60 mV. Pipette resistance was 6–8 MΩ and filled with an internal solution (in mM): 140 K‐gluconate, 10 hydroxyethyl piperazine ethane sulfonic acid, 7 NaCl, 0.5 ethylene glycol tetraacetic acid and 2 Mg‐ATP adjusted to pH 7.4 in current‐clamp mode. Current step was given from −120 to 80 pA with 20‐pA intervals. Electrical signals were digitized and sampled at 10 kHz with Digidata 1322A (Axon Instruments) and Multiclamp 700B amplifier (Molecular Devices) using pCLAMP 10.2 software (Molecular Devices). Raw data were low‐pass filtered at 2 kHz and collected for off‐line analysis at a sampling rate of 10 kHz using pClamp 10.2 software.

*Long‐term potentiation. *Acute brain slices were prepared as aforementioned with slight modification. The 350‐µm‐thick horizontal hippocampal slices were prepared and incubated at room temperature for 1 h before recording. Field excitatory postsynaptic potential (fEPSP) was recorded from CA1 stratum radiatum of the HPC using a glass pipette filled with aCSF (1–3 MΩ) upon electrical stimulation triggered by a concentric bipolar electrode (CBBPE75, FHC, USA) placed in the Schaffer collateral pathway. Evoked fEPSP responses were digitized and sampled at 10 kHz with Digidata 1322A (Axon Instruments) and Multiclamp 700B amplifier (Molecular Devices) using pCLAMP 10.2 software (Molecular Devices). The stimulation intensity was adjusted to obtain fEPSP slopes of 40%–50% to the maximum. Basal fEPSP response was monitored by electrical stimulations at 0.1 Hz. Theta‐burst stimulation (TBS) for inducing LTP consisted of three trains (1.2 s) of five pulses at 100 Hz and pulse width was 0.2 ms with an inter‐burst interval of 200 ms.

### *Ex‐vivo* Calcium Imaging

300‐µm‐thick hippocampal slices were acutely prepared as aforementioned. Imaging was acquired at one frame per second with a 60x water‐immersion objective lens, and a 488‐nm fluorescent imaging filter was utilized for GCaMP6f imaging. CNO (5 µM) was applied for 100 s after baseline stabilization. Fluorescence imaging was acquired and analyzed with Imaging Workbench (Indec Biosystems), and analyzed with ImageJ software (NIH).

### RNA Sequencing

*RNA preparation*. After harvesting fresh brain samples from acutely decapitated mice under anesthesia, we prepared 1 mm‐thick coronal slices using a mouse brain matrix. Then, using a sharp surgical blade, hippocampal and SN tissues according to their anatomical features on ice under a stereomicroscope was dissected (MEB115, Leica, Germany). We extracted total RNA using the easy‐spin Total RNA Extraction Kit (#17 221, iNtRON, Korea) and assessed the RNA concentration with Nanodrop one (Thermo scientific). Sample libraries for sequencing were prepared by the Novaseq Reagent Kit Preparation Guide (Illumina, USA) by Macrogen Inc. (Korea).

### mRNA‐Seq Data

The raw reads from the sequencer to remove low quality and adapter sequence before analysis and aligned the processed reads to the *Mus musculus (mm10)* using HISAT v2.1.0(1) was pre‐processed. HISAT utilizes two types of indexes for alignment (a global, whole‐genome index and tens of thousands of small local indexes). These two types of indexes are constructed using the same BWT (Burrows–Wheeler transform) a graph FM index (GFM) as Bowtie2. Because of its use of these efficient data structures and algorithms, HISAT generates spliced alignments several times faster than Bowtie and BWA widely used. The reference genome sequence of *Mus musculus (mm10)* and annotation data were downloaded from the NCBI. Transcript assembly and abundance estimation using StringTie(2, 3). After alignment, StringTie v2.1.3b was used to assemble aligned reads into transcripts and to estimate their abundance. It provides the relative abundance estimates as Read Count values of transcript and gene expressed in each sample.

### Statistical Analysis of Gene Expression Level

The relative abundances of gene were measured in Read Count using StringTie. We performed the statistical analysis to find differentially expressed genes using the estimates of abundances for each gene in samples. Genes with one more than zeroed Read Count values in the samples were excluded. To facilitate log2 transformation, 1 was added to each Read Count value of filtered genes. Filtered data were log2‐transformed and subjected to RLE normalization. Statistical significance of the differential expression data was determined using nbinomWaldTest using DESeq2 and fold change in which the null hypothesis was that no difference exists among groups. False discovery rate (FDR) was controlled by adjusting p value using Benjamini‐Hochberg algorithm. For DEG set, hierarchical clustering analysis was performed using complete linkage and Euclidean distance as a measure of similarity. The criteria for DEG was FPKM ≥ 1(in more than one group), |fold change| ≥ 1.5, and adjusted p < 0.05 (for SNpc) or raw p < 0.05 (for HPC). GO analysis was performed based on Gene Set Enrichment Analysis (GSEA; http://www.gsea‐msigdb.org/gsea/msigdb/mouse/annotate.jsp) with FDR q value < 0.05.

### MCHR1 shRNA Synthesis

For gene‐silencing Mchr1, we chose three candidate target sequences from coding sequence of mouse Mchr1 (NM_145 132) gene: **1)** 5′‐GCA CAA GGA GTG TCT CCT ACA‐3′, **2)** 5′‐GCA ACG TCC CTG ACA TCT TCA‐3′, **3)** 5′‐GCC TCA ATC CCT TTG TGT ACA‐3′. We prepared MCHR1‐shRNA candidates whose sequences of complementary oligomers were as follows: **1)** 5′‐TGC ACA AGG AGT GTC TCC TAC ATT CAA GAG ATG TAG GAG ACA CTC CTT GTG CTT TTT TC‐3′ and 3′‐TCG AGA AAA AAG CAC AAG GAG TGT CTC CTA CAT CTC TTG AAT GTA GGA GAC ACT CCT TGT GCA‐5′, **2)** 5′‐TGC AAC GTC CCT GAC ATC TTC ATT CAA GAG ATG AAG ATG TCA GGG ACG TTG CTT TTT TC‐3′ and 3′‐TCG AGA AAA AAG CAA CGT CCC TGA CAT CTT CAT CTC TTG AAT GAA GAT GTC AGG GAC GTT GCA‐5′, and **3)** 5′‐TGC CTC AAT CCC TTT GTG TAC ATT CAA GAG ATG TAC ACA AAG GGA TTG AGG CTT TTT TC‐3′ and 3′‐TCG AGA AAA AAG CCT CAA TCC CTT TGT GTA CAT CTC TTG AAT GTA CAC AAA GGG ATT GAG GCA‐5′ (Table S3, Supporting Information). To test the knockdown efficiency of MCHR1‐shRNA candidates, we obtained GFP‐tagged Mchr1 (NM_145 132) ORF clone from Origene (#MG219644). The knockdown efficiency was tested by quantitative RT‐PCR (qPCR) with cDNA from HEK293T cell line (Korean Cell Line Bank) which were transfected with the GFP‐tagged Mchr1 full clone and shRNA vectors. qPCR was performed with QuantStudio 1 Real‐Time PCR machine (Applied Biosystems, USA). qPCR was carried out using SYBR Green PCR Master Mix (Applied Biosystems). In brief, reactions were performed in duplicates in a total volume of 10 µL containing 10 pM primer, 40 ng cDNA, and 5 µL power SYBR Green PCR Master Mix. The mRNA level was normalized to that of GAPDH mRNA. Fold‐induction was calculated using the 2^–∆∆Ct^ method.

### Preparation of hDA Neurons from Human iPSCs

The hDA neurons were derived from human iPSCs (Coriell, GM25256) by using STEMdiff Midbrain Neuron Differentiation Kit (STEM CELL tech, #100‐0038) according to manufacturer instructions. Immunocytochemistry was performed for counting the TH‐ and MAP2‐positive cell numbers. qPCR was carried out for measuring the mRNA expression levels of TH, MAP2, TUJ‐1, and GAP43 (Table S3, Supporting Information).

### Statistical Analysis

Statistical analyses were performed using Prism 9 (GraphPad Software, Inc.). Differences between two different groups were analyzed with the two‐tailed Student's unpaired t‐test. For comparison of multiple groups, one‐way analysis of variance (ANOVA) with Tukey's or Dunnett's multiple comparison test, or two‐way ANOVA with Bonferroni's multiple comparison test was assessed. For assessment of the change of a group by a certain intervention, the significance of data was assessed by the two‐tailed Student's paired t‐test or repeated measure one‐way ANOVA. The normality of the distribution of each data set was tested. When the data was not normally distributed, we performed appropriate non‐parametric tests, such as Mann‐Whitney test or Kruskal‐Wallis ANOVA test. For comparisons of two or multiple groups, we also tested if the variances were statistically different across the groups. If the variance was different, appropriate corrections were applied to the statistical tests. *P* < 0.05 was considered to indicate statistical significance throughout the study. The significance level was represented as asterisks (^*^ *p* < 0.05, ^**^ *p* < 0.01, ^***^ *p* < 0.001; *NS*, not significant). Unless otherwise specified, all data were presented as mean ± SEM. No statistical method was used to predetermine sample size. Sample sizes were determined empirically based on previous experiences or reviews of similar experiments in literatures. The numbers of animals used were described in the corresponding figure legends or on each graph. All experiments were done with at least three biological replicates. Experimental groups were balanced in terms of animal age, sex, and weight. Animals were genotyped before experiments, and they were all caged together and treated in the same way. Prior to acupuncture stimulation, virus injection, or drug administration, animals were randomly and evenly allocated to each experimental group. The data analysis of animal experiments was performed by two independent investigators. However, investigators were not blinded to outcome assessments. Detailed statistical information was listed in Table S1 (Supporting Information).

## Conflict of Interest

The authors declare no conflict of interest.

## Supporting information

Supporting Information

Supplemental Table 1

## Data Availability

The data that supports the findings of this study is available in the supplementary material of this article.
