# Supplementary material for: Central Role of Hypothalamic Circuits for Acupuncture's Anti‐Parkinsonian Effects
Source: Adv Sci (Weinh). 2024 Aug 9;11(43):2403245. doi: 10.1002/advs.202403245 (PMC11578310; doi:10.1002/advs.202403245)
Supplement: Supplementary file 1 — Supporting Information [file ADVS-11-2403245-s002.pdf]

ADVANCED SCIENCE
Open Access

Supporting Information

for *Adv. Sci.*, DOI 10.1002/advs.202403245

Central Role of Hypothalamic Circuits for Acupuncture's Anti-Parkinsonian Effects

Ju-Young Oh, Hyowon Lee, Sun-Young Jang, Hyunjin Kim, Geunhong Park, Almas Serikov, Jae-Hwan Jang, Junyeop Kim, Seulkee Yang, Moonsun Sa, Sung Eun Lee, Young-Eun Han, Tae-Yeon Hwang, Sharon Jiyoon Jung, Hee Young Kim, Seung Eun Lee, Soo-Jin Oh, Jeongjin Kim, Jeongyeon Kim, Jongpil Kim, Thomas J. McHugh, C. Justin Lee, Min-Ho Nam and Hi-Joon Park**

Supporting Information

Central role of hypothalamic circuits for acupuncture's anti-parkinsonian effects

Ju-Young Oh†, Hyowon Lee†, Sun-Young Jang, Hyunjin Kim, Geunhong Park, Almas Serikov, Jae-Hwan Jang, Junyeop Kim, Seulkee Yang, Moonsun Sa, Sung Eun Lee, Young-Eun Han, Tae-Yeon Hwang, Sharon Jiyoon Jung, Hee Young Kim, Seung Eun Lee, Soo-Jin Oh, Jeongjin Kim, Jeongyeon Kim, Jongpil Kim, Thomas J. McHugh, C. Justin Lee, Min-Ho Nam, Hi-Joon Park**

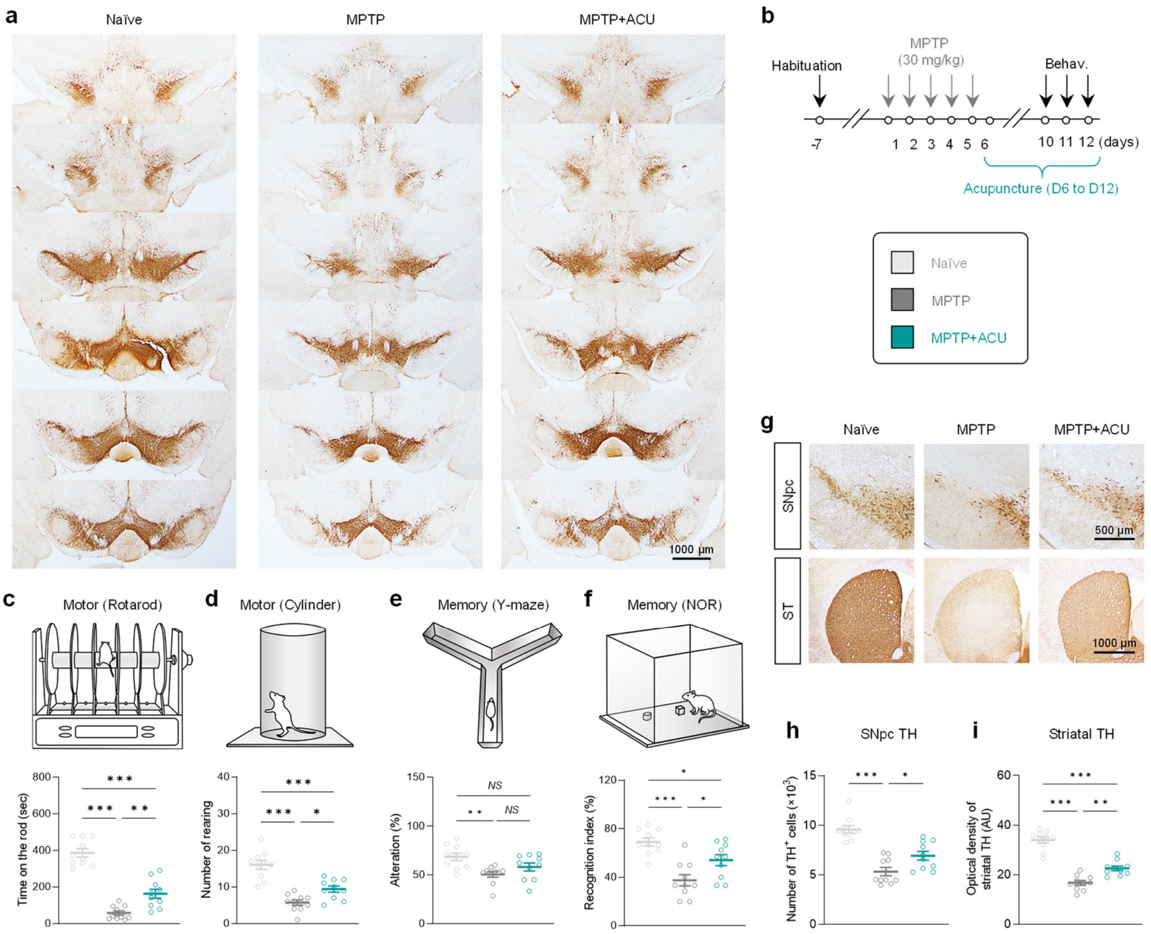

Figure S1. Representative images of serially sectioned SNpc tissues stained with TH antibody and therapeutic effects of acupuncture starting after MPTP treatment. a) TH-positive neurons from the rostral to the caudal portions of the SNpc. b) Timeline of experiments to investigate the therapeutic effect of acupuncture which started after MPTP administration (post-ACU). c-f) Assessment of motor and memory functions. Post-ACU treatment for one week after induction of the MPTP model has a therapeutic effect for motor dysfunction (One-way ANOVA, n = 10 per group; for rotarod: $F_{2,27} = 66.2$, $p < 0.001$; for cylinder: $F_{2,27} = 29.6$, $P < 0.001$; *post-hoc* Tukey's test: $^{***}p < 0.001$, $^{**}p < 0.01$, $^{*}p < 0.05$) and memory impairment (Kruskal-Wallis ANOVA for Y-maze, n = 10 per group: $H_3 = 9.547$, $P = 0.008$; One-way ANOVA for NOR, n = 10 per group: $F_{2,27} = 14.1$, $p < 0.001$; *post-hoc* Tukey's test: $^{***}p < 0.001$, $^{**}p < 0.01$, $^{*}p < 0.05$, *NS*, not significant). g) Representative images of TH expression in SNpc and striatum in MPTP model with or without post-ACU. h,i) Quantification of the number of TH-positive neurons and optical density of striatal TH expression. Post-ACU treatment partially but significantly restored the expression of TH level in the SNpc and striatum (H, Kruskal-Wallis ANOVA for SNpc, n = 10 per group: $H_3 = 20.20$, $p < 0.001$; I, One-way ANOVA for striatum, n = 10 per group: $F_{2,27} = 69.7$, $p < 0.001$; *post-hoc* Tukey's test: $^{***}p < 0.001$, $^{**}p < 0.01$, $^{*}p < 0.05$). Data are shown as mean ± SEM.

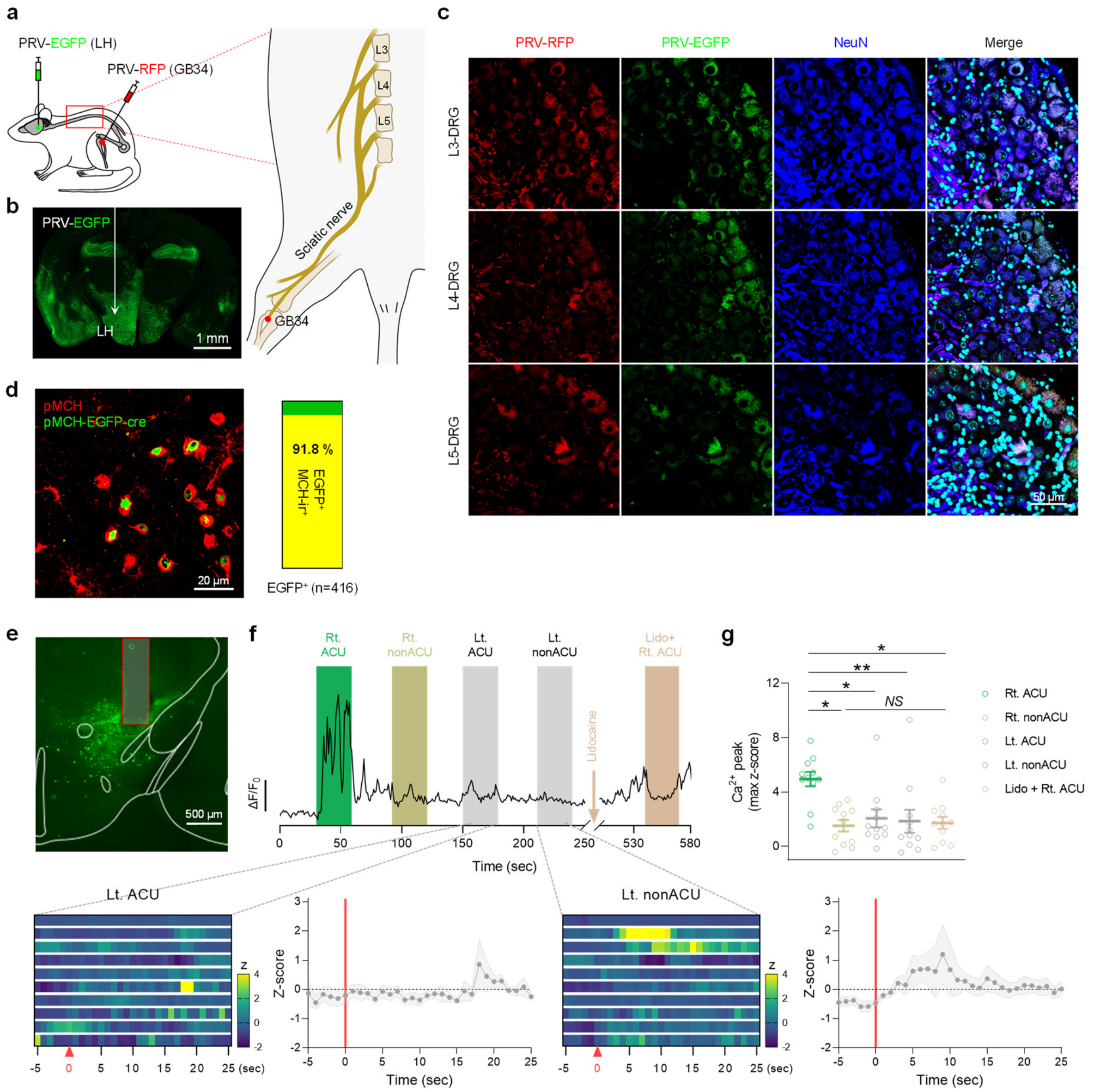

Figure S2. The trans-synaptic retrograde labeling for visualization of neural pathways from hindlimb acupoint GB34 to the LH/ZI. a) Schematic diagram depicting the injection strategy with double retrograde PRVs. PRV-CMV-RFP and PRV-CMV-EGFP were injected into the GB34 and LH, respectively. b) Representative immunofluoresent image demonstrating the PRV-EGFP infection in the LH c) Representative confocal images of DRG (L3 to L5) demonstrating double labeled neurons with PRV-RFP and PRV-EGFP. d) Left, the representative confocal image of pMCH-immunoreactivity and EGFP which is expressed by AAV_{DJ}-pMCH-EGFP-cre virus injection into the mouse LH. Right, Quantification of co-expression of pMCH-EGFP-cre and pMCH (N = 4 mice, n = 416 cells) reveals that EGFP expression was specifically restricted to pMCH-immunolabeled neurons (91.8%, n = 335 cells). e) GRIN lens position and GCaMP6f expression in the LH. f) Top, a representative trace of Ca^{2+} signal of a pMCH neuron upon various acupuncture stimulations. Bottom, heatmaps displaying Ca^{2+} signal of each pMCH neuron and averaged traces of Ca^{2+} signal of pMCH neurons. g) Quantification of Ca^{2+} peak displayed by a scatter plot. The peak Ca^{2+} signals were significantly increased only by the acupuncture treatment at right GB34. (Kruskal-Wallis ANOVA, n = 11 per group; $H_5 = 16.23$, $p = 0.003$; *post-hoc* Dunnett's test: $^{**}p < 0.01$, $^{*}p < 0.05$, *NS*, not significant). Data are shown as mean ± SEM.

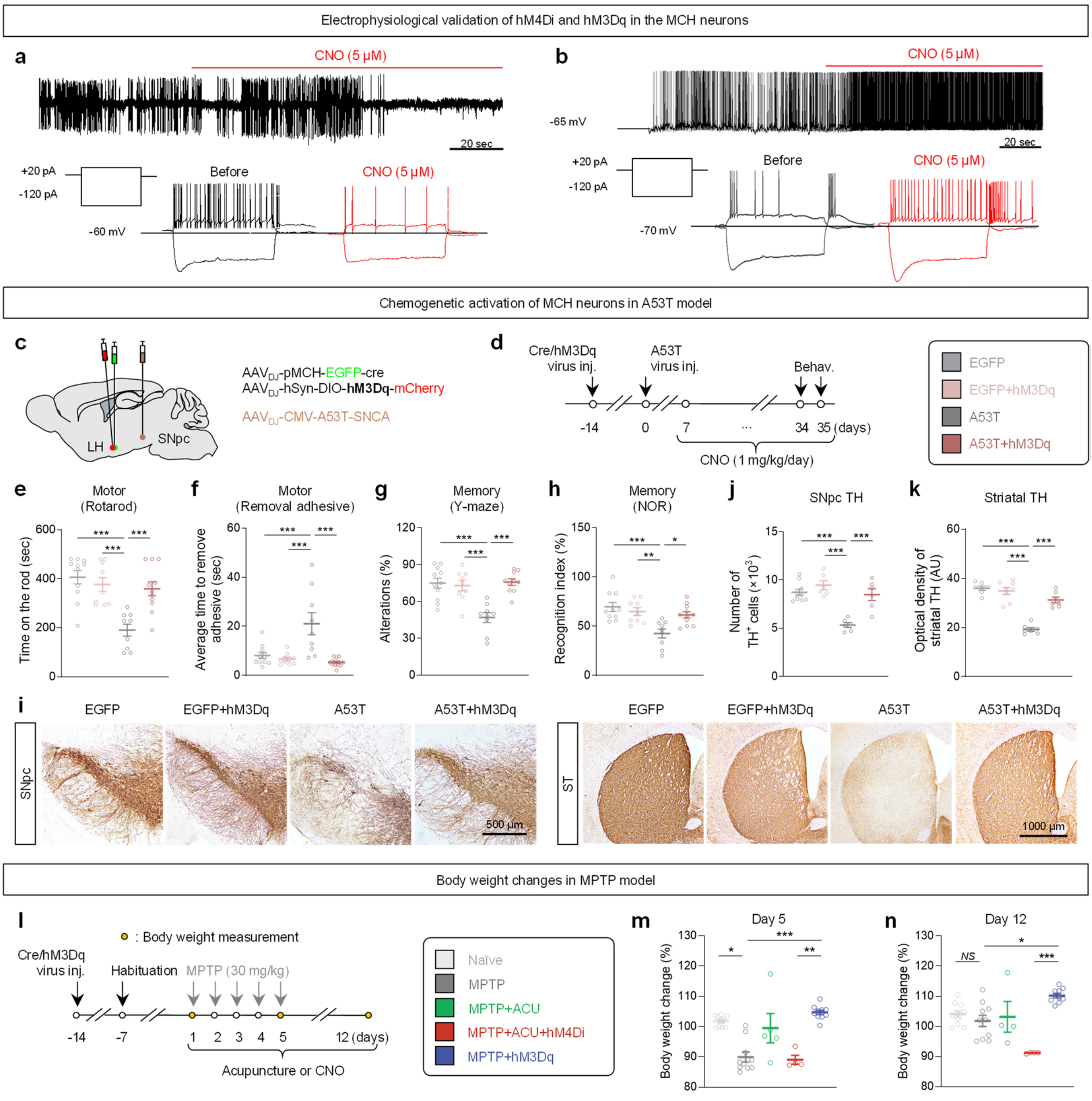

Figure S3. Activation of MCH neurons alleviates parkinsonian motor and memory deficits in two different PD mouse models. a) Electrophysiological validation of hM4Di. Top, cell-attached patch clamp recording of spontaneous action potential before and after bath application of CNO (5 µM). Bottom, current clamp recording of action potential induced by current injection (+20 pA). b) Electrophysiological validation of hM3Dq. Top, current clamp recording of spontaneous firing. Bottom, current clamp recording of action potential induced by current injection (+20 pA). c) Schematic diagram depicting viral strategy for testing chemogenetic approach in A53T model. d) Experimental timeline of chemogenetic activation of MCH neurons in A53T model. e-h) Assessment of motor and memory function by rotarod test, adhesive removal test, Y-maze test, and novel object recognition test. Chemogenetic activation of $\text{pMCH}^{LH/ZI}$ neurons alleviated the motor dysfunction (e,f; Kruskal-Wallis ANOVA for rotarod: $H_4 = 17.92$, $p < 0.001$; One-way ANOVA for removal adhesive: $F_{3,36} = 10.4$, $P < 0.001$; *post-hoc* Tukey's test: $^{***}p < 0.001$, $^{**}p < 0.01$, $^{*}p < 0.05$) and memory deficits (g,h; One-way ANOVA; for Y-maze: $F_{3,35} = 12.7$, $p < 0.001$; for NOR: $F_{3,35} = 8.10$, $p < 0.001$; *post-hoc* Tukey's test: $^{***}p < 0.001$, $^{**}p < 0.01$, $^{*}p < 0.05$) in A53T model. i, Representative images of TH staining in the SNpc and striatum. j,k) Quantification of the number of TH-positive neurons and optical density of striatal TH expression. The number of TH-positive cells

in the SNpc and the optical density of TH-positive dopaminergic fibers in the striatum were restored by chemogenetic activation of $pMCH^{LH/ZI}$ neurons in the A53T model (One-way ANOVA; for SNpc: $F_{3, 24} = 17.5$, $p < 0.001$; for striatum: $F_{3, 25} = 46.4$, $p < 0.001$; *post-hoc* Tukey's test: $^{***}p < 0.001$). l) Timeline of experiments for MPTP model. m,n) Acupuncture and chemogenetic activation of MCH neurons reverses MPTP-induced body weight loss at day 5 (Kruskal-Wallis ANOVA; for day 5: $H_5 = 26.60$, $p < 0.001$; for day 12: $H_5 = 20.42$, $p < 0.001$; *post-hoc* Tukey's test: $^{***}p < 0.001$, $^{**}p < 0.01$, $^{*}p < 0.05$). Data are shown as mean ± SEM.

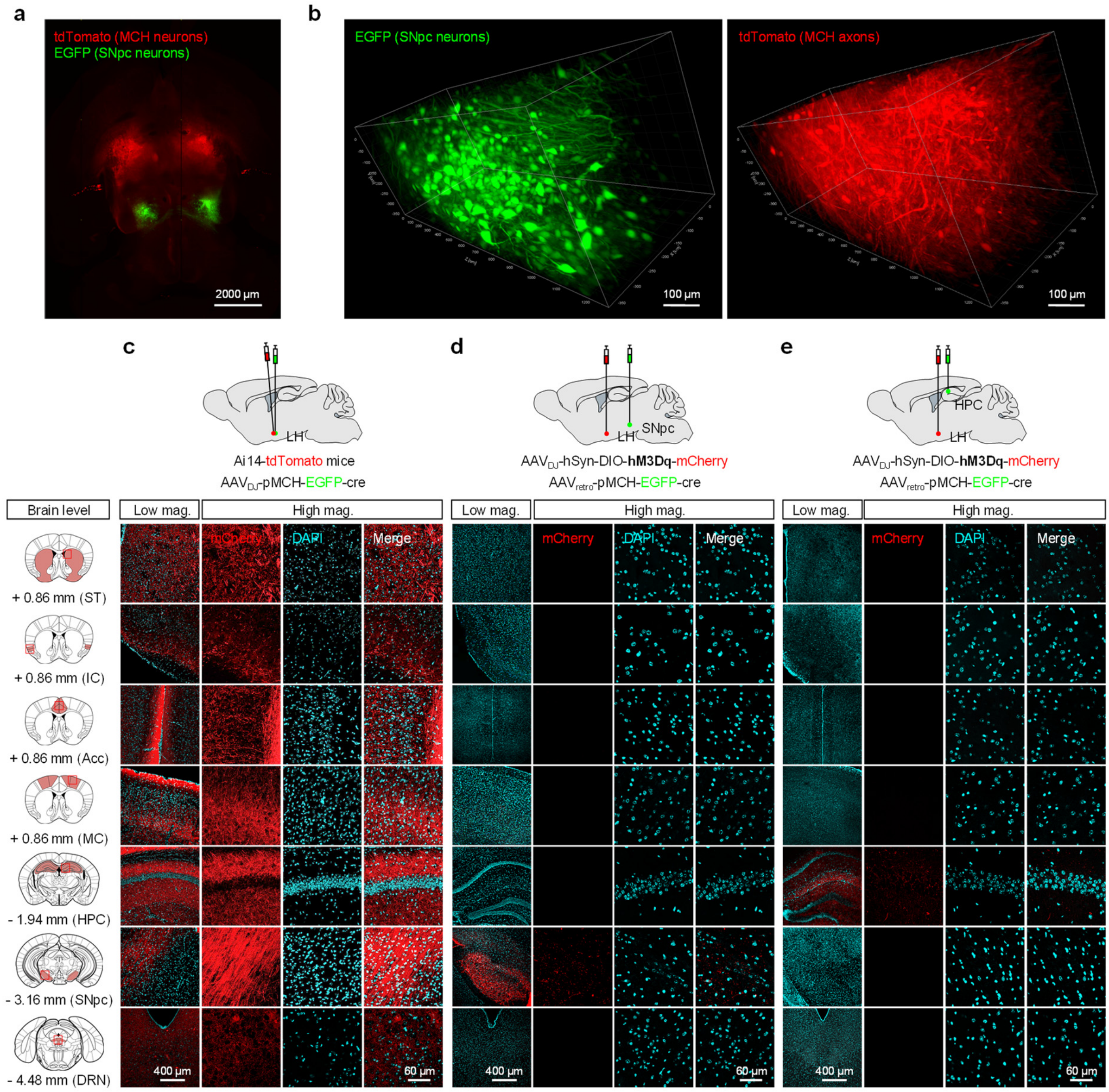

Figure S4. Anatomical analysis of MCH neuronal projections and Intrinsic electrophysiological properties of MCH$^{LH/ZI\rightarrow SNpc}$ and MCH$^{LH\rightarrow HPC}$ neurons. a) Three-dimensional rendering of a cleared mouse brain showing brain-wide injection patterns of MCH neurons labeled by tdTomato and EGFP (SNpc neurons) in the Ai14 (Rosa26-Stop-tdTomato) mouse. b) SNpc neurons (EGFP) co-expressed with axons of MCH neurons projected from LH (tdTomato) in the SNpc. The merged image is displayed in Figure 4b (middle). c-e) Confocal images of striatum (ST), insular cortex (IC), anterior cingulate cortex (ACC), motor cortex (MC), hippocampus (HPC), substantia nigra pars compacta (SNpc), dorsal raphe nucleus (DRN) in the mice of tdTomato-labeling within MCH$^{LH/ZI}$ neurons (c), MCH$^{LH/ZI\rightarrow SNpc}$ neurons (d), and MCH$^{LH\rightarrow HPC}$ neurons (e).

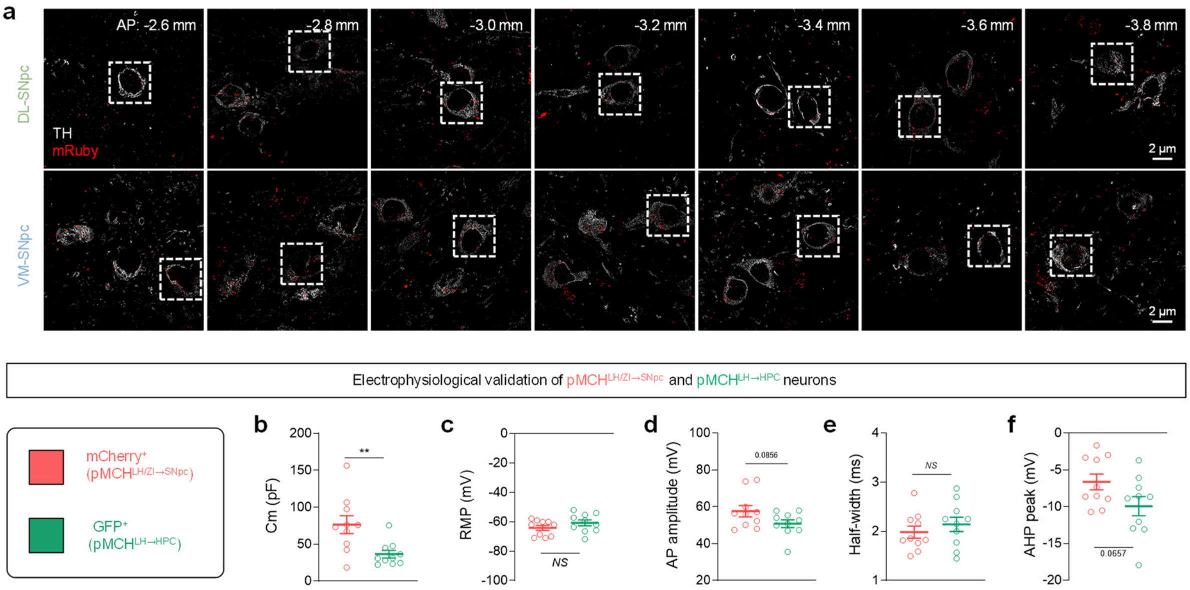

Figure S5. MCH neuronal projections are originated from discrete neuronal subpopulations. a) Lattice-SIM image of synaptophysin::mRuby near the TH-positive neurons in the SNpc. b) Membrane capacitance (two-tailed unpaired t-test, n = 10 per group; $t_{18} = 3.053$, $p = 0.007$). c) Resting membrane potential (two-tailed unpaired t-test, n = 10 per group; $t_{18} = 1.303$, $p = 0.2088$). d) AP amplitude (two-tailed unpaired t-test, n = 10 per group; $t_{18} = 1.819$, $p = 0.0856$). e) AP threshold AP half-width (two-tailed unpaired t-test, n = 10 per group; $t_{18} = 0.8436$, $p = 0.4100$). f) After-hyperpolarization peak (two-tailed unpaired t-test, n = 10 per group; $t_{18} = 1.960$, $p = 0.0657$). Data are shown as mean ± SEM.

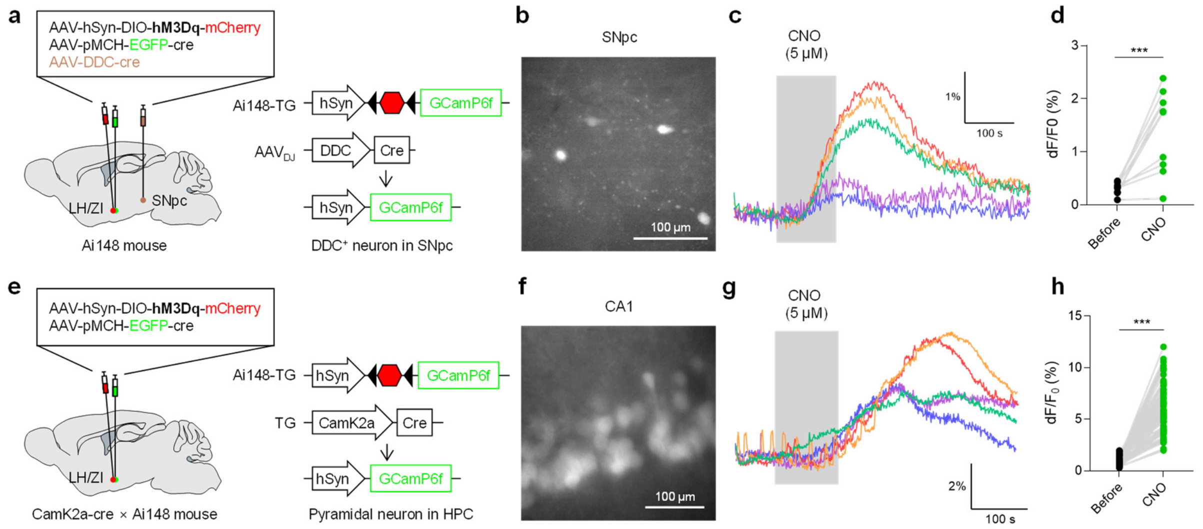

Figure S6. Functional connection of the discrete neural projections of MCH neurons from LH and ZI to SNpc and HPC. a,e) Schematic diagram of *ex vivo* Ca^{2+} imaging of SNpc dopaminergic neurons (a) and CA1 hippocampal pyramidal neurons (e) upon hM3Dq-mediated chemogenetic activation of $MCH^{LH/ZI}$ neurons. b,f) Representative images of GCaMP6f expression in SNpc (b) and CA1 HPC (f). c,g) Ca^{2+} signal traces of SNpc dopaminergic neurons (c) and CA1 hippocampal pyramidal neurons (g). d,h) Quantification of Ca^{2+} peak upon chemogenetic activation of MCH neurons in each region (two-tailed unpaired t-test; SNpc, n = 10 per group, $t_{18} = 4.593$, $p < 0.001$; CA1, n = 101 per group, $t_{200} = 21.52$, $p < 0.001$).

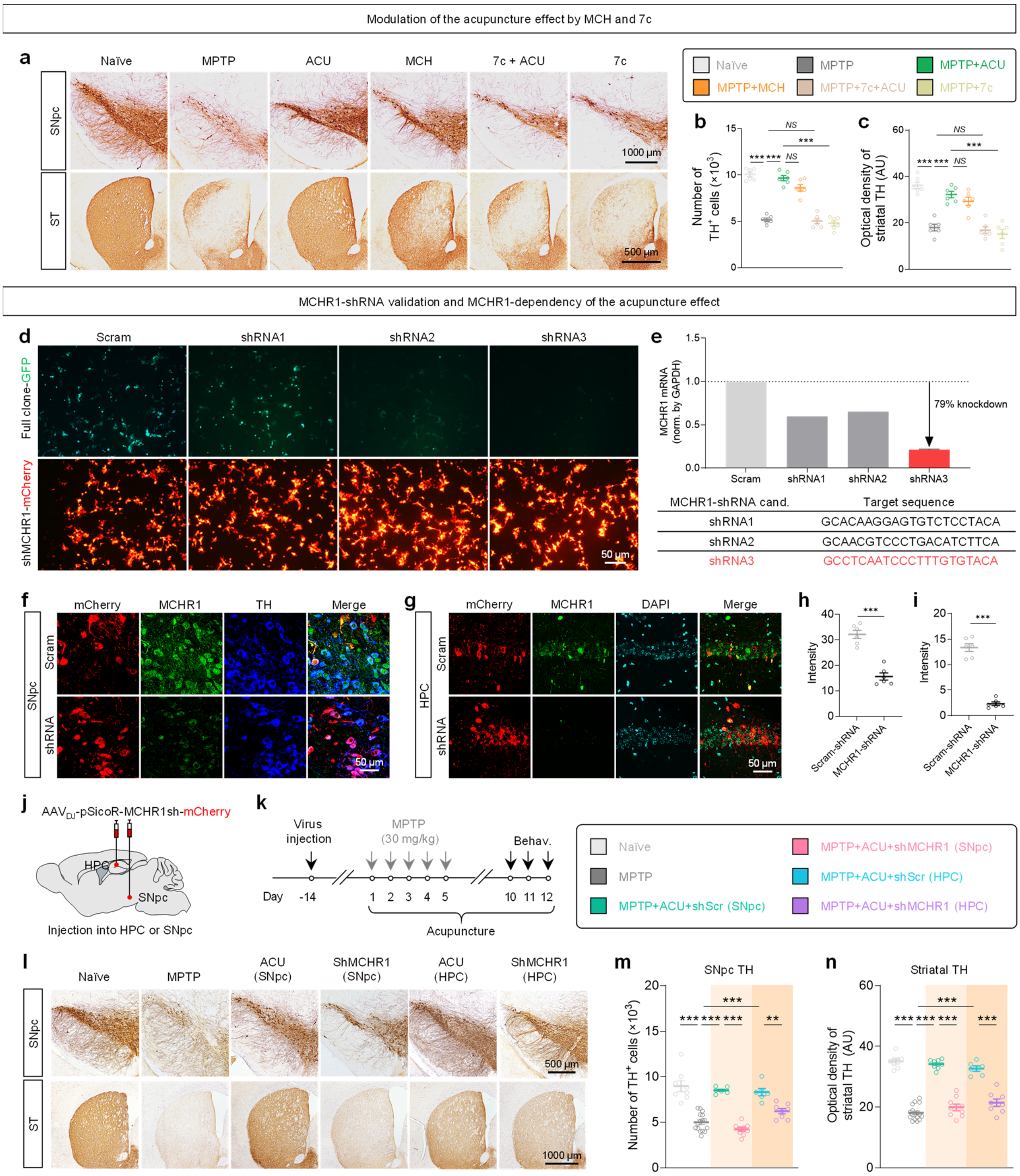

Figure S7. MCHR1 gene-silencing blocks the acupuncture effect. a) Representative images of TH expression in the SNpc and striatum. b) Numbers of TH-positive dopaminergic neurons in the SNpc (One-way ANOVA, n = 6 per group: $F_{5, 30} = 78.9$, $p = 0.855$; *post-hoc* Tukey's test: $^{***}p < 0.001$, *NS*, not significant). c) Quantification of optical density of striatal TH (One-way ANOVA, n = 6 per group: $F_{5, 30} = 32.4$, $p = 0.962$; *post-hoc* Tukey's test: $^{***}p < 0.001$, *NS*, not significant). d) Representative fluorescent images of HEK293T cells displaying the expression of shRNA candidates (mCherry) and the reduced expression of MCHR1 full clone (GFP) 24 h after co-transfection of MCHR1 full clone and the shRNA candidates. e) Top, *in vitro* knockdown efficiency of MCHR1-shRNA candidates. Relative levels of MCHR1 mRNA expression were quantified by normalizing with GAPDH mRNA. Knockdown efficacy was most pronounced by transfection with shRNA3, showing ~79% decrease compared to Scram non-knockdown control. Bottom, the target sequences of shRNA candidates. f-i) In vivo

knockdown efficiency of MCHR1-shRNA3 in SNpc and HPC, validated by immunohistochemistry. Compared with the Scrambled shRNA, shRNA significantly reduced the intensity of MCHR1 expression in the SNpc (h) and HPC (i) (two-tailed unpaired t-test, n = 6 per group; for SNpc: $t_{10} = 7.782$; $p < 0.0001$; for HPC: $t_{10} = 13.07$; $p < 0.0001$). j) Schematic diagram of the location of the AAV_{DJ}-pSicoR-MCHR1sh-mCherry virus injection in the SNpc and HPC. k) Timeline of experiments for *in vivo* gene-silencing of MCHR1 in the MPTP model. l) Representative images of TH staining in the SNpc and striatum. m,n) Quantification of the number of TH-positive neurons and optical density of striatal TH expression. The number of TH-positive cells in SNpc and the optical density of TH-positive dopaminergic fibers in the striatum were significantly blocked by MCHR1 gene-silencing in the SNpc, but less by gene-silencing in the HPC (One-way ANOVA; for SNpc: $F_{5, 47} = 35.0$, $p < 0.001$; for striatum: $F_{5, 50} = 89.5$, $p < 0.001$; *post-hoc* Tukey's test: $^{***}p < 0.001$, $^{**}p < 0.01$). Data are shown as mean ± SEM.

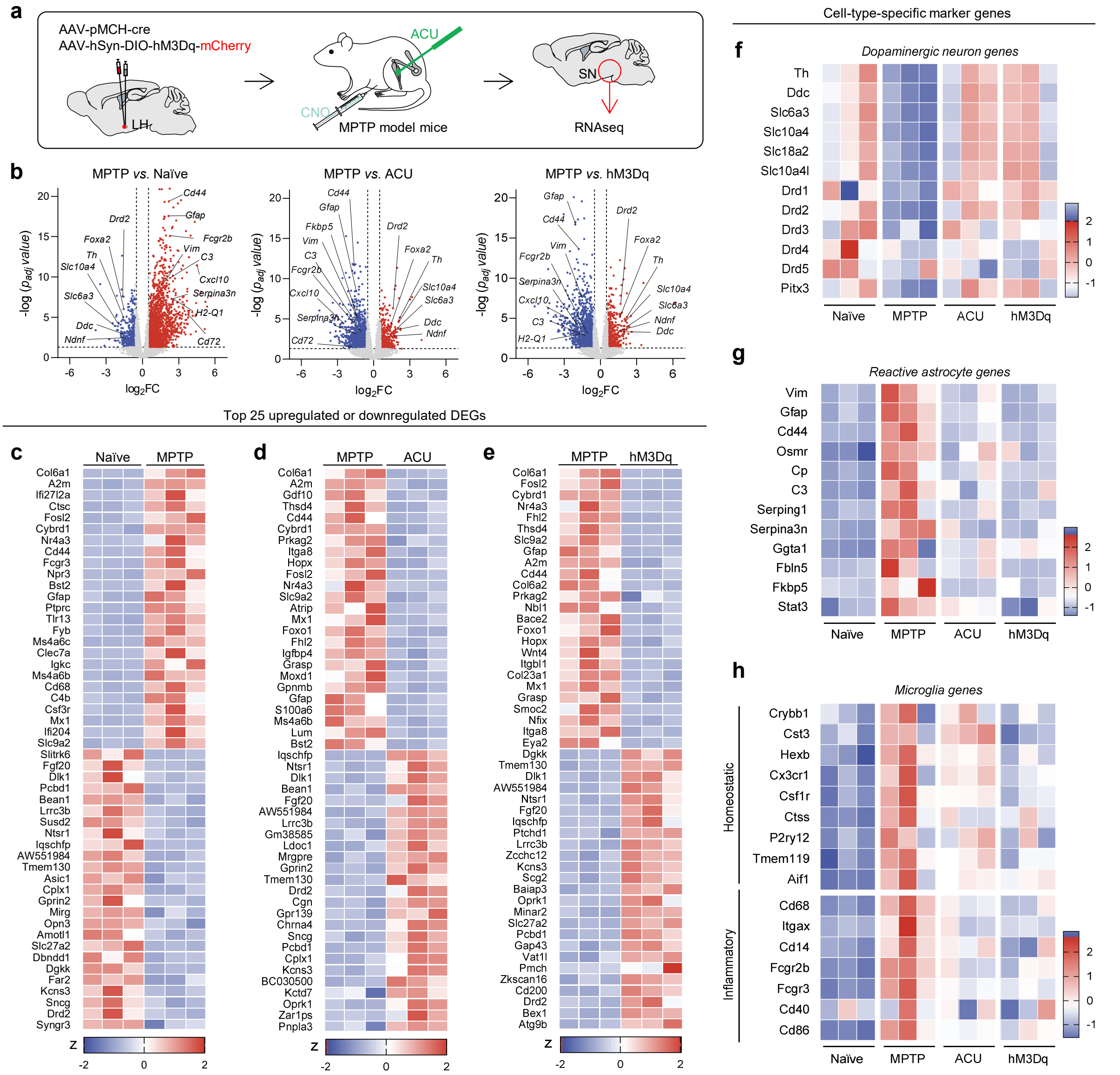

Figure S8. Transcriptomic change in the SNpc by acupuncture or chemogenetic activation of MCH neurons in MPTP model mice. A) Schematic diagram of SNpc tissue preparation for RNA-seq. B) Volcano plot displaying the transcriptomic differences in the SNpc between MPTP and Naïve (left), between MPTP and ACU (middle), and MPTP and hM3Dq groups (right). Upregulated DEGs are marked in red, while downregulated DEGs are marked in blue. DEGs were defined with the criteria of $p_{adj} < 0.05$ and $|FC| > 1.5$. c) Top 25 upregulated and downregulated SNpc DEGs by MPTP treatment with the highest statistical significance. DEGs were identified by three criteria: $p_{adj} < 0.05$, $|FC| \geq 1.5$, and FPKM (in any group) > 1.0. d) Top 25 downregulated and upregulated SNpc DEGs by acupuncture treatment in MPTP model with the highest statistical significance. E) Top 25 downregulated and upregulated SNpc DEGs by hM3Dq-mediated chemogenetic activation of MCH neurons in MPTP model with the highest statistical significance. F-h) Heatmap showing enriched genes in specific cell types such as DA neurons (f), reactive astrocytes (g), and microglia (h) in the SNpc.

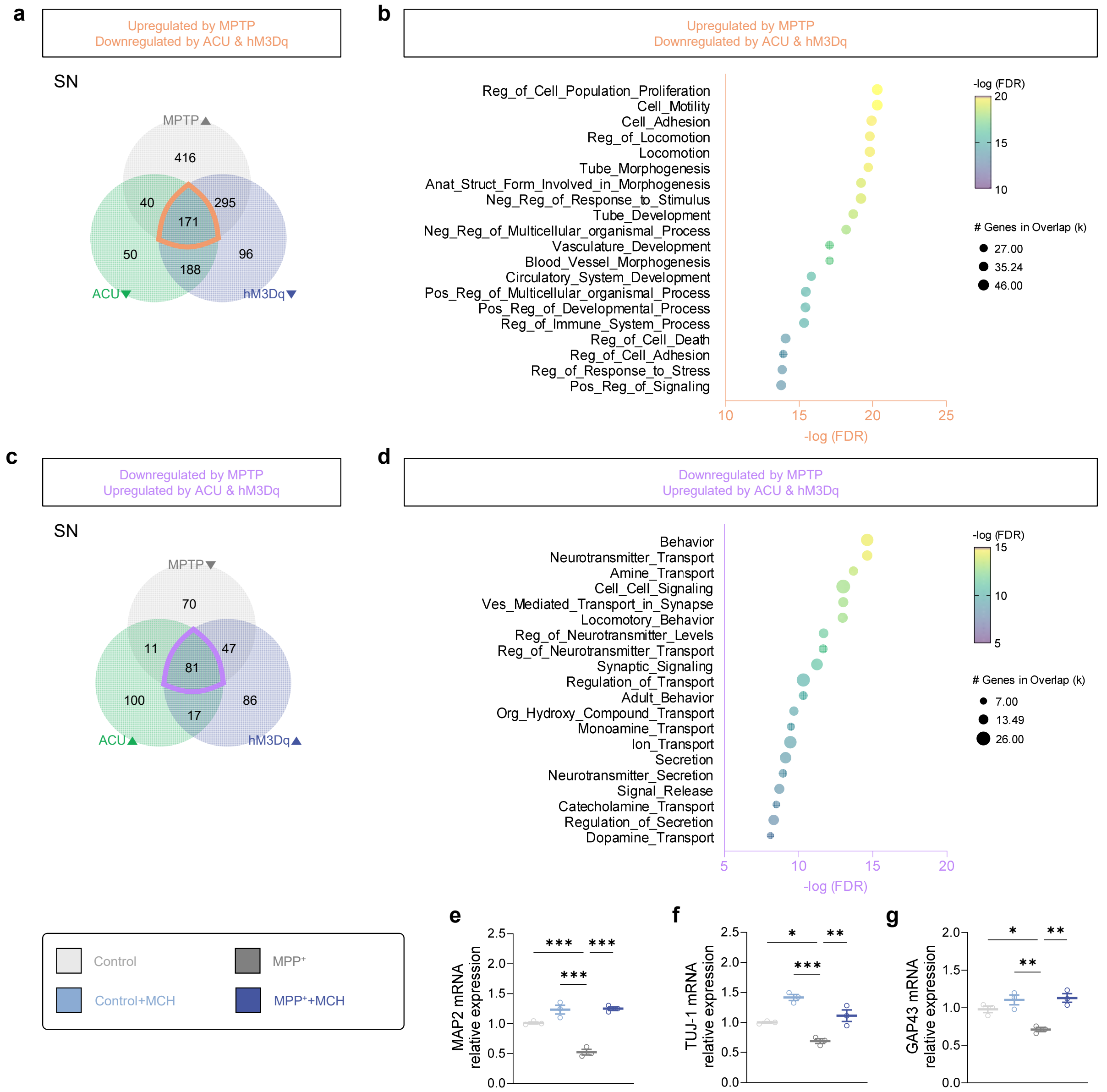

Figure S9. GO analysis of SNpc DEGs. A) Venn diagrams depicting the intersection of upregulated DEGs by MPTP (MPTP-up) and downregulated DEGs by either acupuncture or hM3Dq-mediated activation (ACU-down or hM3Dq-down). B) Top 20 gene ontology (GO) terms analyzed from the intersection of MPTP-up, ACU-down, and hM3Dq-down (marked in orange in (a)) with the highest statistical significance. c) Venn diagrams depicting the intersection of downregulated DEGs by MPTP (MPTP-down) and upregulated DEGs by either acupuncture or hM3Dq-mediated activation (ACU-up or hM3Dq-up). Note that many of the terms are related to the cellular reaction upon neuroinflammation. d) Top 20 gene ontology (GO) terms analyzed from the intersection of MPTP-down, ACU-up, and hM3Dq-up (marked in violet in (c)) with the highest statistical significance. Note that many of the terms are related to the synthesis, transport, signaling, and secretion of amine neurotransmitter (especially, dopamine). e-g) mRNA expressions of MAP2 (e), TUJ-1 (f), and GAP43 (g) assessed by RT-qPCR at 5 days after treatment of MCH along with MPP$^+$ (One-way ANOVA; for MAP2: $F_{3,8} = 51.49$, $p < 0.001$; for TUJ-1: $F_{3,8} = 26.63$, $p = 0.321$; for GAP43: $F_{3,8} = 14.55$, $p = 0.8207$; *post-hoc* Tukey's test: $^{***}p < 0.001$, $^{**}p < 0.01$, $^{*}p < 0.05$). Data are shown as mean ± SEM.

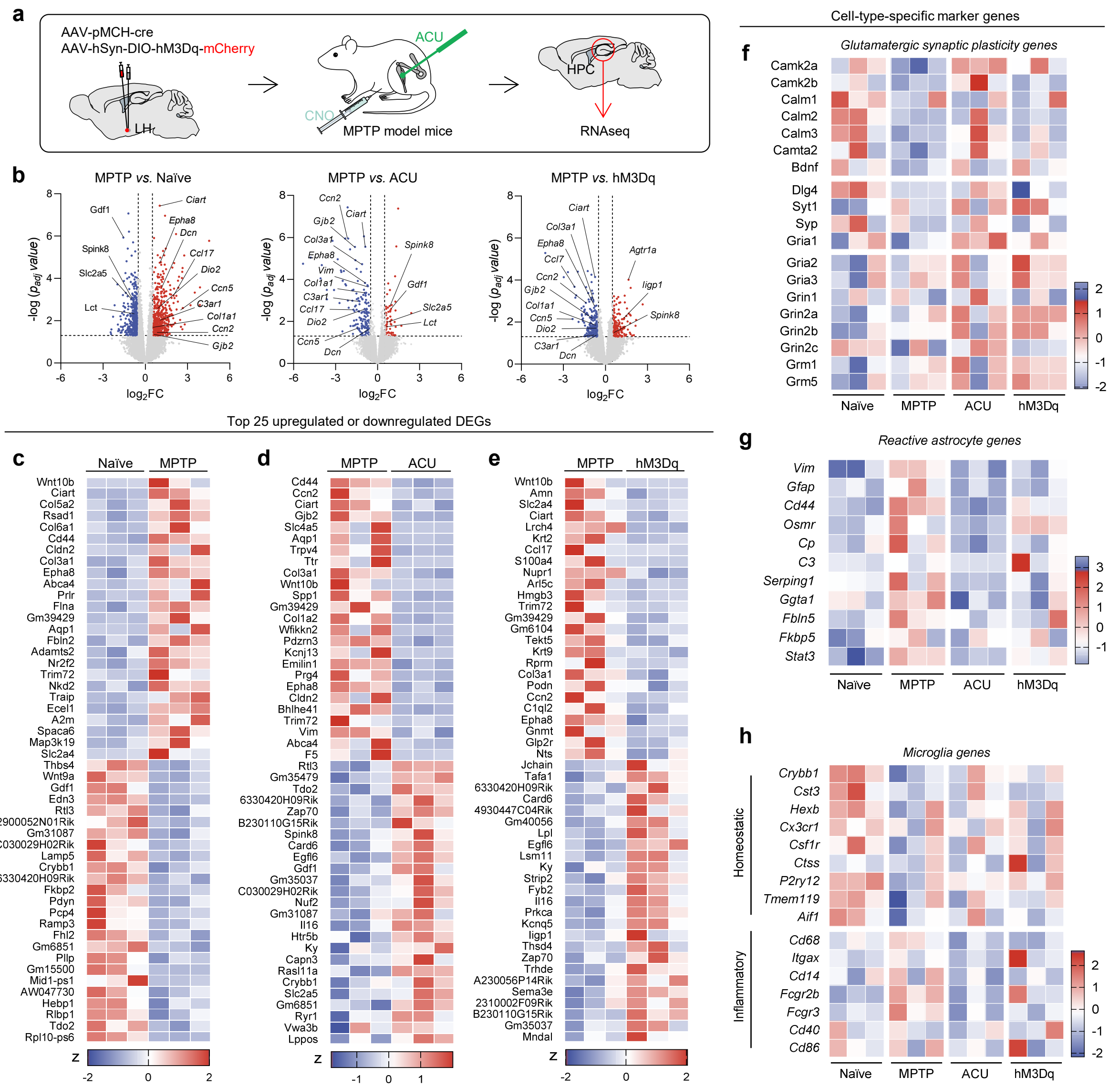

Figure S10. Transcriptomic change in the HPC by acupuncture or chemogenetic activation of MCH neurons in MPTP model mice. a) Schematic diagram of HPC tissue preparation for RNA-seq. b) Volcano plot displaying the transcriptomic differences in the hippocampus between MPTP and Naïve (left), between MPTP 8 and ACU (middle), and MPTP and hM3Dq groups (right). DEGs were defined with the criteria of $p < 0.05$ and $|FC| > 1.5$. c) Top 25 upregulated and downregulated hippocampal DEGs by MPTP treatment with the highest statistical significance. DEGs were identified by three criteria: $p < 0.05$, $|FC| \geq 1.5$, and FPKM (in any group) > 1.0. d) Top 25 downregulated and upregulated hippocampal DEGs by acupuncture treatment in MPTP model with the highest statistical significance. e) Top 25 downregulated and upregulated hippocampal DEGs by hM3Dq-mediated chemogenetic activation of MCH neurons in MPTP model with the highest statistical significance. f-h) Heatmap showing enriched genes in specific cell types such as glutamatergic synapses (f), reactive astrocytes (g), and microglia (h).

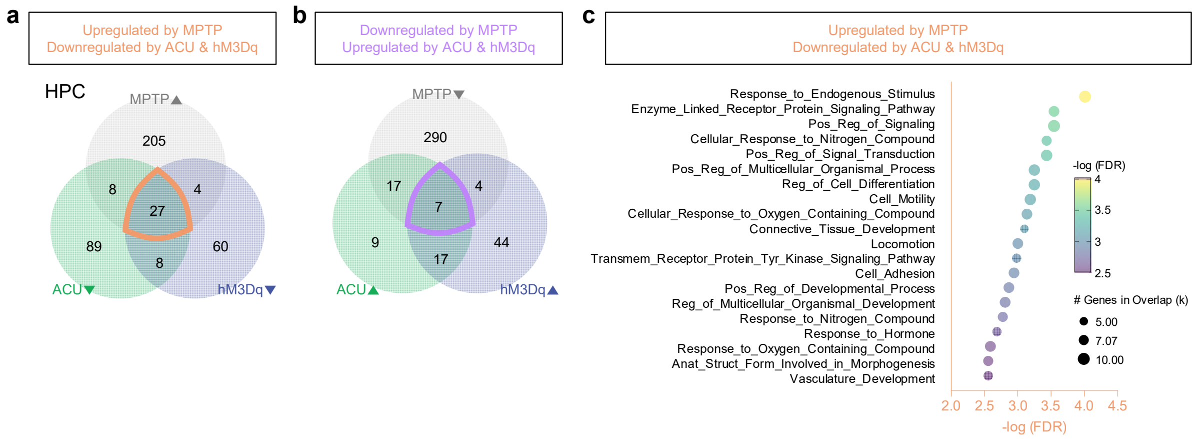

Figure S11. GO analysis of HPC DEGs. a) Venn diagrams depicting the intersection of upregulated DEGs by MPTP (MPTP-up) and downregulated DEGs by either acupuncture or hM3Dq-mediated activation (ACU-down or hM3Dq-down). b) Venn diagrams depicting the intersection of downregulated DEGs by MPTP (MPTP-down) and upregulated DEGs by either acupuncture or hM3Dq-mediated activation (ACU-up or hM3Dq-up). c) Top 20 gene ontology (GO) terms analyzed from the intersection of MPTP-up, ACU-down, and hM3Dq-down (marked in orange in (a)) with the highest statistical significance. Due to the small number of genes in the intersection of MPTP-down, ACU-up, and hM3Dq-up (marked in violet in (b)), GO analysis was not available.

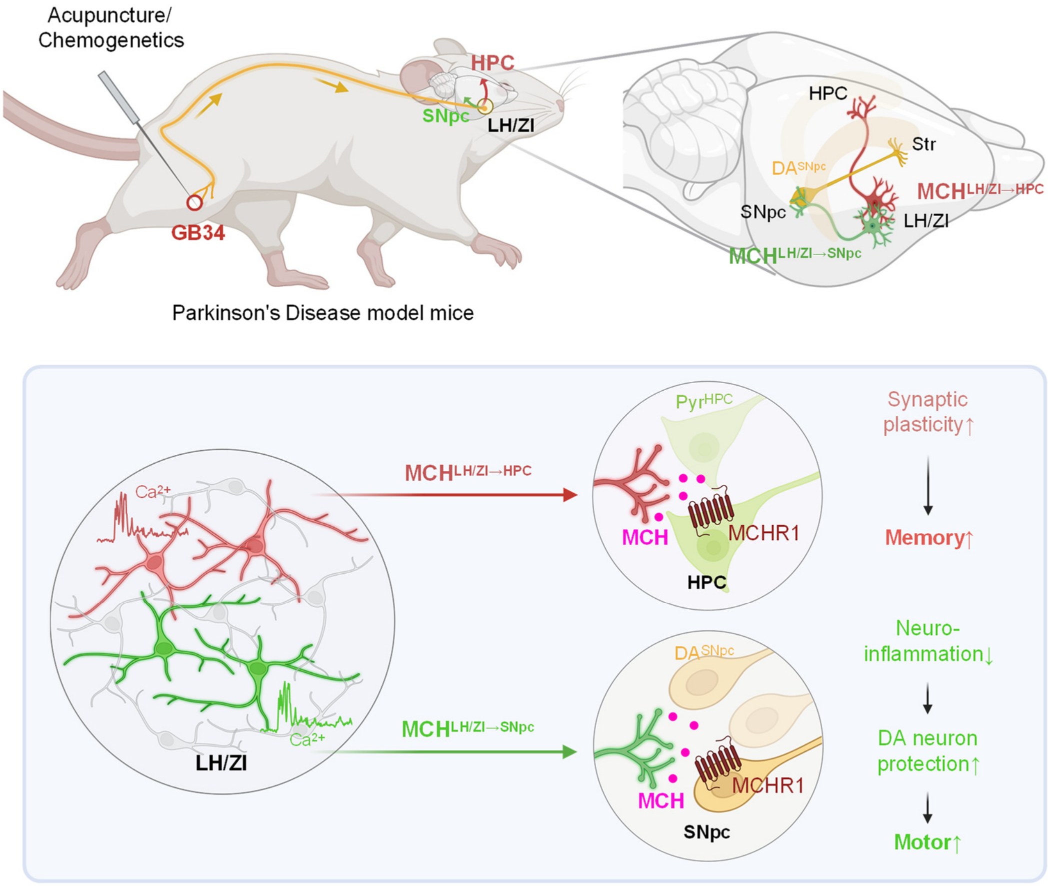

Figure S12. Schematic diagram of the mechanism underlying the acupuncture effect on motor and non-motor symptoms in the PD mouse model. Acupuncture stimulation at a hindlimb acupoint GB34 activates the sensory afferents which are connected to the LH/ZI, leading to activation of MCH neurons in the LH and ZI (MCH$^{LH/ZI}$ neurons). Likewise, chemogenetic activation of the sensory afferents at GB34 activates the MCH$^{LH/ZI}$ neurons. MCH$^{LH/ZI}$ neurons project to HPC and SNpc which originate from distinct subpopulations. Activation of MCH$^{LH\rightarrow HPC}$ projections results in enhancement of hippocampal synaptic plasticity which causes memory improvement in the PD mouse model. On the other hand, activation of MCH$^{LH/ZI\rightarrow SNpc}$ projections exerts a strong anti-inflammatory effect through MCHR1-dependent manner, causing the protection of nigrostriatal DA neurons and improving the motor function in the PD mouse model. In summary, activations of MCH$^{LH\rightarrow HPC}$ and MCH$^{LH/ZI\rightarrow SNpc}$ projections are critical for alleviating the memory and motor dysfunction by acupuncture, respectively. Image was created with Biorender.com.

Table S1. The detailed information about statistical analyses.

Figure No.	Result from statistical analysis
Figure 1f	Naïve (397.2 ± 8.3, n = 16), MPTP (179.3 ± 17.0, n = 16), MPTP+ACU (367.7 ± 13.1, n = 16), MPTP+Lido+ACU (222.8 ± 17.2, n = 12), SCNx+MPTP+ACU (39.2 ± 12.6, n = 5), MPTP+nonACU (164.2 ± 10.8, n = 10)<br>The data were not normally distributed.<br>Kruskal-Wallis ANOVA test with Dunn's multiple comparison test<br>H (6) = 59.65, p < 0.001
Figure 1g	Naïve (24.8 ± 1.5, n = 18), MPTP (6.7 ± 0.7, n = 18), MPTP+ACU (21.7 ± 0.9, n = 18), MPTP+Lido+ACU (8.3 ± 0.9, n = 14), SCNx+MPTP+ACU (5.6 ± 1.0, n = 8), MPTP+nonACU (5.9 ± 1.0, n = 14)<br>One-way ANOVA with Tukey's multiple comparisons test<br>F (5, 84) = 70.56, p < 0.001
Figure 1i	Naïve (9.5 ± 0.2, n = 14), MPTP (5.4 ± 0.2, n = 14), MPTP+ACU (8.8 ± 0.3, n = 14), MPTP+Lido+ACU (5.7 ± 0.2, n = 10), SCNx+MPTP+ACU (5.3 ± 0.2, n = 10), MPTP+nonACU (5.5 ± 0.3, n = 11)<br>One-way ANOVA with Tukey's multiple comparisons test<br>F (5, 67) = 54.91, p < 0.001
Figure 1j	Naïve (39.0 ± 1.6, n = 14), MPTP (19.5 ± 1.4, n = 14), MPTP+ACU (33.2 ± 1.3, n = 14), MPTP+Lido+ACU (22.6 ± 1.3, n = 10), SCNx+MPTP+ACU (21.3 ± 1.3, n = 10), MPTP+nonACU (19.1 ± 1.0, n = 16)<br>One-way ANOVA with Tukey's multiple comparisons test<br>F (5, 72) = 41.20, p < 0.001
Figure 1k	Naïve (72.5 ± 3.3, n = 11), MPTP (49.3 ± 3.0, n = 11), MPTP+ACU (69.5 ± 3.6, n = 11), MPTP+Lido+ACU (41.3 ± 2.7, n = 11), SCNx+MPTP+ACU (34.9 ± 3.7, n = 11), MPTP+nonACU (44.9 ± 3.3, n = 11)<br>One-way ANOVA with Tukey's multiple comparisons test<br>F (5, 60) = 25.49, p < 0.001
Figure 1l	Naïve (73.6 ± 2.8, n = 11), MPTP (46.7 ± 3.0, n = 11), MPTP+ACU (70.6 ± 3.1, n = 11), MPTP+Lido+ACU (43.8 ± 2.8, n = 11), SCNx+MPTP+ACU (37.4 ± 3.6, n = 11), MPTP+nonACU (44.8 ± 2.8, n = 11)<br>One-way ANOVA with Tukey's multiple comparisons test<br>F (5, 48) = 18.93, p < 0.001
Figure 2e	Rt. ACU (5.0 ± 0.5, n = 11), Rt. nonACU (1.5 ± 0.4, n = 11), Lido + Rt. AU (1.7 ± 0.4, n = 11)<br>Repeated-Measure one-way ANOVA with Dunnett's multiple comparisons test (compared with Rt. ACU group)<br>F (2, 20) = 18.92, p < 0.001
Figure 2i	CTL (14.45 ± 1.4, n = 3), CNO (26.90 ± 1.9, n=4)<br>Unpaired t test, p < 0.001
Figure 3b	Naïve (423.1 ± 13.0, n = 19), MPTP (83.3 ± 13.6, n = 15), MPTP+ACU (331.6 ± 19.0, n = 9), MPTP+hM4Di+ACU (61.2 ± 7.6, n = 13), MPTP+hM3Dq (355.2 ± 18.4, n = 21)<br>One-way ANOVA with Tukey's multiple comparisons test<br>F (4, 72) = 112.8, p < 0.001
Figure 3c	Naïve (19.8 ± 1.2, n = 19), MPTP (3.8 ± 0.8, n = 15), MPTP+ACU (21.1 ± 1.4, n = 9), MPTP+hM4Di+ACU (3.9 ± 0.4, n = 13), MPTP+hM3Dq (22.7 ± 1.8, n = 21)<br>One-way ANOVA with Tukey's multiple comparisons test<br>F (4, 72) = 47.05, p < 0.001
Figure 3e	Naïve (8.5 ± 0.3, n = 11), MPTP (5.2 ± 0.3, n = 11), MPTP+ACU (8.6 ± 0.2, n = 11), MPTP+hM4Di+ACU (5.0 ± 0.2, n = 11), MPTP+hM3Dq (8.4 ± 0.5, n = 11)<br>The data were not normally distributed.<br>Kruskal-Wallis ANOVA test with a Dunn's multiple comparison test, p < 0.001<br>H (5) = 37.32, p < 0.001
Figure 3f	Naïve (32.7 ± 0.5, n = 10), MPTP (13.4 ± 0.5, n = 15), MPTP+ACU (26.9 ± 0.8, n = 12), MPTP+hM4Di+ACU (13.3 ± 0.7, n = 12), MPTP+hM3Dq (28.7 ± 0.9, n = 10)<br>One-way ANOVA with Tukey's multiple comparisons test<br>F (4, 54) = 177, p < 0.001
Figure 3g	Naïve (75.1 ± 2.1, n = 19), MPTP (42.7 ± 3.0, n = 15), MPTP+ACU (75.6 ± 3.4, n = 9), MPTP+hM4Di+ACU (34.3 ± 2.1, n = 13), MPTP+hM3Dq (74.0 ± 1.8, n = 21)<br>One-way ANOVA with Tukey's multiple comparisons test<br>F (4, 72) = 73.2, p < 0.001
Figure 3h	Naïve (74.7 ± 1.3, n = 19), MPTP (35.3 ± 3.3, n = 17), MPTP+ACU (72.3 ± 1.7, n = 9), MPTP+hM4Di+ACU (33.9 ± 3.3, n = 15), MPTP+hM3Dq (69.9 ± 1.7, n = 23)<br>One-way ANOVA with Tukey's multiple comparisons test<br>F (4, 78) = 72.45, p < 0.001
Figure 3j	Naïve (168.7 ± 12.9, n = 10), MPTP (101.8 ± 9.2, n = 10), MPTP+ACU (183.7 ± 22.4, n = 10), MPTP+hM4Di+ACU (99.2 ± 16.2, n = 10), MPTP+hM3Dq (175.9 ± 25.6, n = 10)<br>One-way ANOVA with Tukey's multiple comparisons test<br>F (4, 45) = 5.213, p = 0.0015
Figure 4e	Multiple unpaired two-tailed t-test<br>-2.6 mm: Ventromedial (16.2 ± 0.8, n = 6), Dorsolateral (8.9 ± 1.4, n = 6), p = 0.001088<br>-2.8 mm: Ventromedial (16.0 ± 1.9, n = 6), Dorsolateral (12.1 ± 1.5, n = 6), p = 0.140115<br>-3.0 mm: Ventromedial (13.9 ± 2.4, n = 6), Dorsolateral (11.2 ± 2.0, n = 6), p = 0.404874<br>-3.2 mm: Ventromedial (16.5 ± 2.6, n = 6), Dorsolateral (10.3 ± 1.3, n = 6), p = 0.060982<br>-3.4 mm: Ventromedial (17.0 ± 0.8, n = 6), Dorsolateral (10.3 ± 1.1, n = 6), p = 0.000603<br>-3.6 mm: Ventromedial (10.2 ± 1.5, n = 6), Dorsolateral (12.4 ± 2.1, n = 6), p = 0.416505<br>-3.8 mm: Ventromedial (13.6 ± 1.9, n = 6), Dorsolateral (9.9 ± 1.5 n = 6), p = 0.160308
Figure 4k	mCherry^{+} (-33.4 ± 1.6, n = 10), GFP^{+} (-28.9 ± 0.8, n = 10)<br>Unpaired two-tailed t-test, p = 0.023
Figure 4l	Multiple unpaired two-tailed t-test<br>-120 pA: mCherry^{+} (0.0 ± 0.0, n = 10), GFP^{+} (0.0 ± 0.0, n = 10), p = 0.135

	-100 pA: mCherry+ (0.0 ± 0.0, n = 10), GFP+ (0.0 ± 0.0, n = 10), p = 0.135<br>-80 pA: mCherry+ (0.0 ± 0.0, n = 10), GFP+ (0.0 ± 0.0, n = 10), p = 0.135<br>-60 pA: mCherry+ (0.0 ± 0.0, n = 10), GFP+ (0.0 ± 0.0, n = 10), p = 0.135<br>-40 pA: mCherry+ (0.0 ± 0.0, n = 10), GFP+ (0.0 ± 0.0, n = 10), p = 0.135<br>-20 pA: mCherry+ (0.0 ± 0.0, n = 10), GFP+ (0.0 ± 0.0, n = 10), p = 0.135<br>0 pA: mCherry+ (0.6 ± 0.4, n = 10), GFP+ (0.1 ± 0.1, n = 10), p = 0.269<br>20 pA: mCherry+ (6.2 ± 2.3, n = 10), GFP+ (2.3 ± 1.1, n = 10), p = 0.141<br>40 pA: mCherry+ (10.9 ± 3.9, n = 10), GFP+ (5.6 ± 1.8, n = 10), p = 0.233<br>60 pA: mCherry+ (15.2 ± 5.2, n = 10), GFP+ (8.7 ± 2.9, n = 10), p = 0.294<br>80 pA: mCherry+ (18.4 ± 5.6, n = 10), GFP+ (10.7 ± 3.8, n = 10), p = 0.271<br>100 pA: mCherry+ (21.7 ± 5.8, n = 10), GFP+ (12.6 ± 4.5, n = 10), p = 0.230<br>120 pA: mCherry+ (21.9 ± 5.4, n = 10), GFP+ (13.8 ± 5.0, n = 10), p = 0.286<br>140 pA: mCherry+ (24.5 ± 5.6, n = 10), GFP+ (14.3 ± 5.0, n = 10), p = 0.190<br>160 pA: mCherry+ (26.4 ± 5.8, n = 10), GFP+ (11.7 ± 4.1, n = 10), p = 0.054<br>180 pA: mCherry+ (27.3 ± 6.0, n = 10), GFP+ (8.4 ± 2.9, n = 10), p = 0.010<br>200 pA: mCherry+ (29.3 ± 6.5, n = 10), GFP+ (6.7 ± 2.3, n = 10), p = 0.004<br>220 pA: mCherry+ (30.4 ± 7.0, n = 10), GFP+ (5.6 ± 1.7, n = 10), p = 0.003
Figure 4m	mCherry+ (-120, 3.1 ± 0.8, n = 10; -100, 3.0 ± 1.0, n = 10; -80, 2.9 ± 1.0, n = 10; -60, 2.7 ± 1.0, n = 10; -40, 2.0 ± 1.1, n = 10; -20, 1.4 ± 1.0, n = 10; 0, 0.1 ± 0.1, n = 10; 20, 0.0 ± 0.0, n = 10; 40, 0.0 ± 0.0, n = 10; 60, 0.0 ± 0.0, n = 10; 80, 0.0 ± 0.0, n = 10; 100, 0.0 ± 0.0, n = 10; 120, 0.0 ± 0.0, n = 10; 140, 0.0 ± 0.0, n = 10; 160, 0.0 ± 0.0, n = 10; 180, 0.0 ± 0.0, n = 10; 200, 0.0 ± 0.0, n = 10; 220, 0.0 ± 0.0, n = 10), GFP+ (-120, 1.1 ± 0.4, n = 10; -100, 0.9 ± 0.3, n = 10; -80, 0.8 ± 0.3, n = 10; -60, 0.5 ± 0.2, n = 10; -40, 0.1 ± 0.1, n = 10; -20, 0.1 ± 0.1, n = 10; 0, 0.0 ± 0.0, n = 10; 20, 0.0 ± 0.0, n = 10; 40, 0.0 ± 0.0, n = 10; 60, 0.0 ± 0.0, n = 10; 80, 0.0 ± 0.0, n = 10; 100, 0.0 ± 0.0, n = 10; 120, 0.0 ± 0.0, n = 10; 140, 0.0 ± 0.0, n = 10; 160, 0.0 ± 0.0, n = 10; 180, 0.0 ± 0.0, n = 10; 200, 0.0 ± 0.0, n = 10; 220, 0.0 ± 0.0, n = 10)<br>Two-way ANOVA with Sidak's multiple comparisons test<br>Interaction, F (17, 306) = 3.604, p < 0.0001; Injected currents, F (1.235, 22.22) = 10.33, p = 0.0025; Neuron subtype, F (1, 18) = 3.997, p = 0.0609
Figure 5b	Naïve (398.7 ± 19.0, n = 13), MPTP (25.2 ± 3.9, n = 14), MPTP+ACU (370.2 ± 18.7, n = 11), MPTP+hM4Di+ACU (48.4 ± 7.5, n = 9), MPTP+hM3Dq (215.9 ± 16.8, n = 8)<br>One-way ANOVA with Tukey's multiple comparisons test<br>F (4, 50) = 156, p < 0.001
Figure 5c	Naïve (17.9 ± 1.5, n = 16), MPTP (4.8 ± 0.3, n = 14), MPTP+ACU (17.2 ± 0.5, n = 11), MPTP+hM4Di+ACU (6.6 ± 0.6, n = 9), MPTP+hM3Dq (18.8 ± 0.9, n = 8)<br>One-way ANOVA with Tukey's multiple comparisons test<br>F (4, 53) = 46.08, p < 0.001
Figure 5d	Naïve (79.0 ± 1.9, n = 16), MPTP (35.1 ± 3.0, n = 13), MPTP+ACU (80.6 ± 2.1, n = 11), MPTP+hM4Di+ACU (66.8 ± 2.9, n = 9), MPTP+hM3Dq (34.3 ± 3.7, n = 8)<br>One-way ANOVA with Tukey's multiple comparisons test<br>F (4, 52) = 72.8, p < 0.001
Figure 5e	Naïve (70.2 ± 2.4, n = 16), MPTP (35.5 ± 3.1, n = 19), MPTP+ACU (69.5 ± 2.4, n = 11), MPTP+hM4Di+ACU (57.5 ± 1.2, n = 9), MPTP+hM3Dq (38.1 ± 2.5, n = 8)<br>One-way ANOVA with Tukey's multiple comparisons test<br>F (4, 58) = 39.11, p < 0.001
Figure 5g	Naïve (9.2 ± 0.4, n = 10), MPTP (5.6 ± 0.2, n = 10), MPTP+ACU (9.2 ± 0.4, n = 10), MPTP+hM4Di+ACU (5.9 ± 0.4, n = 9), MPTP+hM3Dq (8.2 ± 0.3, n = 7)<br>One-way ANOVA with Tukey's multiple comparisons test<br>F (4, 41) = 28.7, p < 0.001
Figure 5h	Naïve (32.2 ± 0.6, n = 10), MPTP (16.4 ± 0.8, n = 10), MPTP+ACU (28.0 ± 0.8, n = 10), MPTP+hM4Di+ACU (15.7 ± 0.6, n = 9), MPTP+hM3Dq (24.7 ± 0.9, n = 7)<br>One-way ANOVA with Tukey's multiple comparisons test<br>F (4, 41) = 99.9, p < 0.001
Figure 5j	Naïve (384.3 ± 19.0, n = 16), MPTP (61.6 ± 12.5, n = 14), MPTP+ACU (336.0 ± 24.9, n = 11), MPTP+hM4Di+ACU (199.4 ± 17.9, n = 10), MPTP+hM3Dq (45.4 ± 8.0, n = 10)<br>One-way ANOVA with Tukey's multiple comparisons test<br>F (4, 56) = 79.68, p < 0.001
Figure 5k	Naïve (20.3 ± 0.8, n = 16), MPTP (4.6 ± 0.5, n = 14), MPTP+ACU (17.5 ± 0.7, n = 11), MPTP+hM4Di+ACU (14.2 ± 0.6, n = 10), MPTP+hM3Dq (8.5 ± 1.2, n = 10)<br>One-way ANOVA with Tukey's multiple comparisons test<br>F (4, 56) = 82.8, p < 0.001
Figure 5l	Naïve (76.3 ± 2.6, n = 16), MPTP (34.1 ± 1.8, n = 14), MPTP+ACU (77.6 ± 2.0, n = 11), MPTP+hM4Di+ACU (44.8 ± 2.4, n = 10), MPTP+hM3Dq (67.4 ± 4.1, n = 10)<br>One-way ANOVA with Tukey's multiple comparisons test<br>F (4, 56) = 60.4, p < 0.001
Figure 5m	Naïve (73.3 ± 1.3, n = 16), MPTP (38.1 ± 3.1, n = 15), MPTP+ACU (74.2 ± 1.5, n = 11), MPTP+hM4Di+ACU (39.8 ± 2.1, n = 10), MPTP+hM3Dq (70.0 ± 2.4, n = 10)<br>One-way ANOVA with Tukey's multiple comparisons test<br>F (4, 57) = 67.9, p < 0.001
Figure 5o	Naïve (8.5 ± 0.3, n = 11), MPTP (4.7 ± 0.2, n = 12), MPTP+ACU (7.3 ± 0.3, n = 8), MPTP+hM4Di+ACU (5.6 ± 0.3, n = 10), MPTP+hM3Dq (4.8 ± 0.2, n = 10)<br>The data were not normally distributed.<br>Kruskal-Wallis ANOVA test with a Dunn's multiple comparison test, p < 0.001<br>H (5) = 35.89, p < 0.001
Figure 5p	Naïve (34.2 ± 0.5, n = 11), MPTP (16.0 ± 0.7, n = 12), MPTP+ACU (28.2 ± 1.2, n = 8), MPTP+hM4Di+ACU (20.0 ± 1.2, n = 10), MPTP+hM3Dq (19.3 ± 0.9, n = 10)<br>One-way ANOVA with Tukey's multiple comparisons test

	F (4, 46) = 74.4, p < 0.001
Figure 6b	Naïve (430.8 ± 18.2, n = 6), MPTP (142.7 ± 15.8, n = 6), MPTP+ACU (355.7 ± 25.2, n = 6), MPTP+MCH (274.7 ± 52.2, n = 6), MPTP+7c+ACU (182.5 ± 26.2, n = 6), MPTP+7c (166.8 ± 29.5, n = 6)<br>One-way ANOVA with Tukey's multiple comparisons test<br>F (5, 30) = 14.6, p < 0.001
Figure 6c	Naïve (14.7 ± 1.2, n = 6), MPTP (3.0 ± 1.0, n = 6), MPTP+ACU (12.3 ± 1.0, n = 6), MPTP+MCH (9.0 ± 1.4, n = 6), MPTP+7c+ACU (6.2 ± 1.0, n = 6), MPTP+7c (3.0 ± 0.7, n = 6)<br>One-way ANOVA with Tukey's multiple comparisons test<br>F (5, 30) = 19.8, p < 0.001
Figure 6d	Naïve (75.4 ± 3.8, n = 6), MPTP (50.5 ± 2.1, n = 6), MPTP+ACU (70.8 ± 1.7, n = 6), MPTP+MCH (62.2 ± 2.5, n = 6), MPTP+7c+ACU (52.5 ± 3.3, n = 6), MPTP+7c (46.8 ± 6.9, n = 6)<br>The data were not normally distributed.<br>Kruskal-Wallis ANOVA test with a Dunn's multiple comparison test, p < 0.001<br>H (6) = 25.46, p < 0.001
Figure 6e	Naïve (75.7 ± 3.4, n = 6), MPTP (42.3 ± 5.3, n = 6), MPTP+ACU (70.0 ± 2.5, n = 6), MPTP+MCH (65.6 ± 3.0, n = 6), MPTP+7c+ACU (51.6 ± 2.0, n = 6), MPTP+7c (45.9 ± 4.2, n = 6)<br>The data were not normally distributed.<br>Kruskal-Wallis ANOVA test with a Dunn's multiple comparison test, p < 0.001<br>H (6) = 28.31, p < 0.001
Figure 6g	Naïve (361.9 ± 11.5, n = 13), MPTP (96.3 ± 7.6, n = 16), MPTP+ACU+shScr (SNpc) (321.6 ± 18.9, n = 8), MPTP+ACU+shMCHR1 (SNpc) (189.4 ± 21.9, n = 8), MPTP+ACU+shScr (HPC) (350.4 ± 14.6, n = 8), MPTP+ACU+shMCHR1 (HPC) (339.0 ± 13.8, n = 8)<br>One-way ANOVA with Tukey's multiple comparisons test<br>F (5, 55) = 79.2, p < 0.001
Figure 6h	Naïve (25.2 ± 1.1, n = 13), MPTP (11.4 ± 0.8, n = 15), MPTP+ACU+shScr (SNpc) (22.8 ± 1.0, n = 9), MPTP+ACU+shMCHR1 (SNpc) (16.4 ± 1.1, n = 9), MPTP+ACU+shScr (HPC) (26.1 ± 0.9, n = 8), MPTP+ACU+shMCHR1 (HPC) (24.4 ± 1.4, n = 8)<br>One-way ANOVA with Tukey's multiple comparisons test<br>F (5, 56) = 38.2, p < 0.001
Figure 6i	Naïve (78.5 ± 1.5, n = 13), MPTP (57.3 ± 1.8, n = 16), MPTP+ACU+shScr (SNpc) (71.5 ± 2.7, n = 8), MPTP+ACU+shMCHR1 (SNpc) (62.4 ± 4.5, n = 9), MPTP+ACU+shScr (HPC) (75.2 ± 1.9, n = 8), MPTP+ACU+shMCHR1 (HPC) (59.1 ± 3.8, n = 8)<br>The data were not normally distributed.<br>Kruskal-Wallis ANOVA test with a Dunn's multiple comparison test, p < 0.001<br>H (6) = 37.75, p < 0.001
Figure 6j	Naïve (79.6 ± 3.1%, n = 13), MPTP (49.5 ± 3.1, n = 16), MPTP+ACU+shScr (SNpc) (73.4 ± 5.8, n = 8), MPTP+ACU+shMCHR1 (SNpc) (60.7 ± 4.3, n = 9), MPTP+ACU+shScr (HPC) (77.3 ± 2.8, n = 8), MPTP+ACU+shMCHR1 (HPC) (56.5 ± 2.4, n = 8)<br>One-way ANOVA with Tukey's multiple comparisons test<br>F (5, 56) = 14.2, p < 0.001
Figure 7e	Control (90.3 ± 4.4, n = 6), MPP^+ (23.8 ± 3.9, n = 6), Control+MCH (91.8 ± 5.4%, n = 6), MPP^++MCH (54.8 ± 4.2, n = 6)<br>One-way ANOVA with Tukey's multiple comparisons test<br>F (3, 20) = 51.42, p < 0.001
Figure 7f	Control (98.0 ± 3.8, n = 6), MPP^+ (23.0 ± 3.4, n = 6), Control+MCH (95.3 ± 6.2, n = 6), MPP^++MCH (55.3 ± 3.8, n = 6)<br>One-way ANOVA with Tukey's multiple comparisons test<br>F (3, 20) = 65.05, p < 0.001
Figure 7g	Control (0.9 ± 0.0, n = 3), MPP^+ (0.7 ± 0.0%, n = 3), Control+MCH (1.1 ± 0.0, n = 3), MPP^++MCH (1.0 ± 0.0, n = 3)<br>One-way ANOVA with Tukey's multiple comparisons test<br>F (3, 8) = 39.27, p < 0.001
Figure 7i	Naïve (760.5 ± 43.3, n = 85), MPTP (1457.6 ± 209.4, n = 23), MPTP+ACU (467.2 ± 23.4, n = 89), MPTP+hM3Dq (582.7 ± 41.8, n = 44)<br>One-way ANOVA with Tukey's multiple comparisons test<br>F (3, 237) = 34.6, p < 0.001
Figure 7j	Naïve (125.0 ± 12.3, n = 79), MPTP (550.1 ± 91.7, n = 23), MPTP+ACU (121.9 ± 10.6, n = 89), MPTP+hM3Dq (223.6 ± 25.3, n = 44)<br>One-way ANOVA with Tukey's multiple comparisons test<br>F (3, 231) = 39.82, p < 0.001
Figure 7k	Naïve (474.7 ± 30.9, n = 42), MPTP (885.4 ± 54.2, n = 39), MPTP+ACU (631.5 ± 35.7, n = 46), MPTP+hM3Dq (622.9 ± 34.1, n = 42)<br>The data were not normally distributed.<br>Kruskal-Wallis ANOVA test with Dunn's multiple comparison test<br>H (4) = 40.60, p < 0.001
Figure7l	Naïve (82.8 ± 8.1, n = 42), MPTP (320.2 ± 18.8, n = 39), MPTP+ACU (125.2 ± 9.7, n = 100), MPTP+hM3Dq (206.6 ± 17.9, n = 42)<br>The data were not normally distributed.<br>Kruskal-Wallis ANOVA test with Dunn's multiple comparison test<br>H (4) = 83.21, p < 0.001
Figure 7n	Two-way ANOVA with Tukey's multiple comparisons test<br>Interaction, F (42,1935) = 7.716, p < 0.001; Radius, F (14, 1935) = 328.4, p < 0.001; Group, F (3, 1935) = 110.6, p < 0.001
Figure 7o	Naïve (19.7 ± 1.3, n = 26), MPTP (40.3 ± 2.8, n = 34), MPTP+ACU (23.0 ± 1.3, n = 36), MPTP+hM3Dq (20.1 ± 1.1, n = 40)

	The data were not normally distributed.<br>Kruskal-Wallis ANOVA test with Dunn's multiple comparison test<br>H (4) = 52.46, $p < 0.001$
Figure 7p	Naïve (2.4 ± 0.8, n = 28), MPTP (5.8 ± 2.9, n = 36), MPTP+ACU (2.6 ± 0.9, n = 38), MPTP+hM3Dq (2.9 ± 1.2, n = 42)<br>The data were not normally distributed.<br>Kruskal-Wallis ANOVA test with Dunn's multiple comparison test<br>H (4) = 20.82, $p < 0.001$
Figure 7t	Naïve (138.8 ± 12.2, n = 5), MPP^+ (95.1 ± 7.4, n = 5), MPTP+MCH100 (127.2 ± 19.7, n = 5), MPTP+MCH200 (158.1 ± 18.3, n = 5)<br>One-way ANOVA with Tukey's multiple comparisons test<br>F (3, 16) = 3.016, p = 0.0607

Table S2. The detailed information about virus titers, volumes, and total amount for each experiment.

Virus	Figure	Titer (GC/ml)	Volume (µl)	Total amount (GC)
AAV_{DJ}-pMCH-cre	2 c, g / 3 a / 4 a, f / 7 h / S2 a, b, d, e / S3 a, b, l / S8 a / S9 a /S1 a / S2 a	5.7x10^13	1.5	8.55x10^10
PRV-CAG-EGFP	2 a / S2 a, b	3.0x10^9	1.5	4.5x10^6
PRV-CAG-RFP	S2 a	3.0x10^9	1.5	4.5x10^6
AAV_{DJ}-hSyn-EGFP	4 a	9.17x10^12	1.5	1.38x10^10
AAV_{retro}-hSyn-DIO-mCherry	4 f	2.23x10^13	1.5	3.35x10^10
AAV_{retro}-hSyn-DIO-EGFP	4 f	1.8x10^13	1.5	2.7x10^10
AAV-pMCH-EGFP-cre	S2 b / S3 c / S4 c / S6 a, e / S2 d	5.96x10^13	1.5	8.94x10^10
AAV_{DJ}-hSyn-DIO-hM3Dq-mCherry	3 a / 5 a, f, k, o / 7 h / S3 b, c, l / S4 d / S4 e / S5 b / S6 a, e / S8 a / S9 a /S1 a	8.77x10^12	1.5	1.32x10^10
AAV-DDC-cre	S6 a	8.9x10^13	1.5	1.34x10^11
AAV_{DJ}-hSyn-DIO-hM4Di-mCherry	3 a / 5 a, f, k, o / S3 a, l / S5 b /S1 a	1.66x10^13	1.5	2.49x10^10
AAV_{retro}-pMCH-EGFP-cre	5 a, f, k, o / S4 d, e / S5 b	8.71x10^13	1.5	1.31x10^11
AAV_{DJ}-pSicoR-MCHR1sh-mCherry	6 f / S7 f, j	2.31x10^13	1.5	3.47x10^10
AAV_{DJ}-pSicoR-scr-shRNA-mCherry	6 f / S7 f, j	4.8x10^13	1.5	7.2x10^10
AAV_{retro}-hSyn-hM3Dq-mCherry	2 g	3.53x10^13	1.5	5.3x10^10
AAV_{DJ}-CMV-A53T-SNCA	S3 c	2.42x10^13	1.5	3.63x10^10
AAV_5-hSYn-DIO-synpatophysin-mRuby	4 c / S5 a	7.88x10^12	1.5	1.18x10^13

Table S3. Sequences of oligonucleotides.

shRNA targeting sequence: Mchr1 #1	5’-GCA CAA GGA GTG TCT CCT ACA-3’
shRNA targeting sequence: Mchr1 #2	5’-GCA ACG TCC CTG ACA TCT TCA-3’
shRNA targeting sequence: Mchr1 #3	5’-GCC TCA ATC CCT TTG TGT ACA-3’
qPCR primer for TH Forward	AGG TCT ACA CCA CGC TGA AG
qPCR primer for TH Reverse	TAC TGG GTG CAC TGG AAC AC
qPCR primer for MAP2 Forward	CTG GCA CCC CAC CAA GTT AT
qPCR primer for MAP2 Reverse	CTT CAG GTC TGG CAG TGG TT
qPCR primer for GAP43 Forward	CCG ATG GGG TGG AGA AGA AG
qPCR primer for GAP43 Reverse	GGA GGA CGG CGA GTT ATC AG
qPCR primer for TUJ1 Forward	CAA CGA GGC CTC TTC TCA CA
qPCR primer for TUJ1 Reverse	CAG GCA GTC GCA GTT TTC AC
